# A Hyperflexible Electrode Array for Long‐Term Recording and Decoding of Intraspinal Neuronal Activity

**DOI:** 10.1002/advs.202303377

**Published:** 2023-10-23

**Authors:** Jie Fan, Xiaocheng Li, Peiyu Wang, Fan Yang, Bingzhen Zhao, Jianing Yang, Zhengtuo Zhao, Xue Li

**Affiliations:** ^1^ Center for Excellence in Brain Science and Intelligence Technology Institute of Neuroscience Chinese Academy of Sciences Shanghai 200031 P. R. China

**Keywords:** hyperflexible electrode, intraspinal recording, neural decoding, neural interface, neural trajectory

## Abstract

Neural interfaces for stable access to the spinal cord (SC) electrical activity can benefit patients with motor dysfunctions. Invasive high‐density electrodes can directly extract signals from SC neuronal populations that can be used for the facilitation, adjustment, and reconstruction of motor actions. However, developing neural interfaces that can achieve high channel counts and long‐term intraspinal recording remains technically challenging. Here, a biocompatible SC hyperflexible electrode array (SHEA) with an ultrathin structure that minimizes mechanical mismatch between the interface and SC tissue and enables stable single‐unit recording for more than 2 months in mice is demonstrated. These results show that SHEA maintains stable impedance, signal‐to‐noise ratio, single‐unit yield, and spike amplitude after implantation into mouse SC. Gait analysis and histology show that SHEA implantation induces negligible behavioral effects and Inflammation. Additionally, multi‐unit signals recorded from the SC ventral horn can predict the mouse's movement trajectory with a high decoding coefficient of up to 0.95. Moreover, during step cycles, it is found that the neural trajectory of spikes and low‐frequency local field potential (LFP) signal exhibits periodic geometry patterns. Thus, SHEA can offer an efficient and reliable SC neural interface for monitoring and potentially modulating SC neuronal activity associated with motor dysfunctions.

## Graphical abstract

A nano‐fabricated hyperflexible electrode array enables chronic recording at single‐neuron resolution in mice spinal cord ventral horn with negligible tissue response and motor impairment. Moreover, with this tool, it is possible to precisely decode spinal cord motor signals and reveal the neural dynamics of movement, which sets a foundation for spinal interface and reliable research methods for spinal studies.

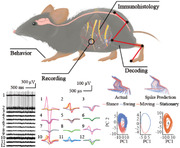

## 1 Introduction

Loss of motor function occurs in many diseases and injuries of the nervous system, inflicting heavy burdens on the patient and society.^[^ ^1^, ^2^ ^]^ Evidence has shown that rehabilitation of normal motor functions could be promoted in these patients to varying degrees by electrically stimulating their propriospinal neural network via the spinal cord (SC) neural interface.^[^ ^3^, ^4^, ^5^, ^6^, ^7^, ^8^ ^]^ In addition, the patient's mobility could be facilitated by external prostheses that are controlled by signals representing the patient's movement intention, as deciphered from movement‐related neuron recordings.^[^ ^9^ ^]^ The SC motor neurons represent the final pathway for motor outputs, and the SC interface could serve to monitor and regulate motor functions. Two major varieties of SC neural interfaces, epidural electrodes, and intraspinal electrodes, have been used to decode movement intention.^[^ ^10^, ^11^, ^12^, ^13^, ^14^ ^]^ Epidural electrodes mainly register aggregated local field potentials, whereas intraspinal electrodes are capable of monitoring both local field potentials and neuronal spikes. Because of their superior spatial and temporal resolution, neuronal spikes are more desirable SC signals for decoding movement intention and for generating signals for fine prosthesis control.

The development of efficient intraspinal interfaces with multiple electrodes faces the following technical challenges. First, the acquisition bandwidth, which is limited by the channel count, remains rather too low to take advantage of potential spatial resolution for single‐unit recording.^[^ ^12^, ^14^ ^]^ Second, fracturing of the electrode due to its mechanical stiffness causes instability of electrical interfacing during long‐term recording,^[^ ^15^, ^16^ ^]^ particularly due to frequent relative movements of SC tissues.^[^ ^17^ ^]^ Furthermore, electrode implants are often significantly more rigid than the SC tissue, causing disparate displacement of the electrode and targeted neurons, causing local tissue damage.^[^ ^16^ ^]^ The mechanical mismatch is known to induce elevated inflammatory responses near the electrodes, leading to glial scar formation, neuron degeneration, and signal degradation at the interface.^[^ ^16^, ^18^ ^]^

Using state‐of‐art nanofabrication technologies, we developed a multichannel SC hyperflexible electrode array (SHEA) for chronic recording and decoding of SC signals. This technology enables the fabrication of a dense 128‐channel electrode array with a spatial resolution of cellular dimensions and a hyperflexible array structure with an electrode bending force comparable to the cellular traction force. Moreover, the 1 µm thickness of SHEA renders a significant reduction of electrode stiffness, resulting in reduced electrode/tissue mechanical mismatch. These properties of SHEA allowed us to collect large amounts of single‐unit signals from stable SC recordings over periods of up to a few months. The prolonged presence of SHEA appeared benign to the SC, as shown by the minimal immune response and scar tissue near the electrode and undetectable interference to the gait behavior of mice. Finally, we demonstrated the quality and usefulness of SHEA‐recorded data in decoding SC ventral horn signals that represent the joint motion of mice during a wheel‐running task and observed running‐related neural trajectories of both spike and local field potential (LFP). Thus, we have developed a high‐density intraspinal electrode array for chronic single‐unit recording that may prove to be useful for future clinical applications of electrical interfaces at the SC level.

## 2 Results

### 2.1 Design and Fabrication of SHEA

**Figure 1:**
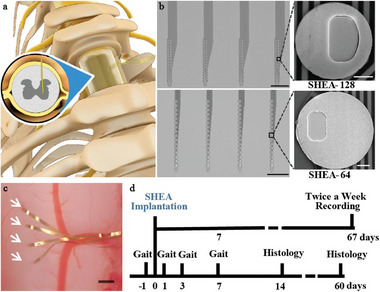
Design of the SHEA and timeline for evaluating mouse behaviors and electrode performance. a) The schematic diagram for SHEA implantation in the SC. The inset depicts the cross‐section of the SC with the SHEA implanted in it. SHEA, spinal cord hyperflexible electrode array. b) Light microscopic images of SHEA‐128 and SHEA‐64 probes and zoomed‐in scanning electronic microscopy images of the electrodes’ recording sites and Via for electrical connection. Scale bars: black, 200 µm; white, 5 µm. c) A light microscopic image of the SHEA vertically implanted in the unilateral SC of an intact mouse spine (white arrows marked the insertion location of 4 shanks). Gold ribbons are the SHEA's body left outside of the SC. Scale bar, 200 µm. d) The timeline for assessing the SHEA performance. Recording (twice a week) was ongoing throughout the 67 days.

To achieve long‐term intraspinal recording, we used state‐of‐art nanotechnology to fabricate the SHEA with two types of electrode patterns, SHEA‐128 and SHEA‐64 (**Figure** **1**a,b; Figure S1, Supporting Information, and Experimental Section). The SHEA‐128 probe had four 5 mm long shanks (thickness 1 µm, mean width 100 µm), each comprising 32 round recording sites (20 µm in diameter) evenly distributed in two columns along the 480 µm distal end of the shank. The SHEA‐64 probe had a similar design, except that the mean width was 50 µm, and 16 round recording sites (30 µm in diameter) were evenly distributed along the 630 µm distal end of the shank. Detailed parameters of the two electrodes are shown in Table S1 (Supporting Information). Compared with traditional rigid SC electrodes,^[^ ^18^, ^19^ ^]^ SHEA has several orders‐of‐magnitudes lower probe volume, a drastically improved flexibility (Figure S2, Supporting Information), and an elevated number of recording sites, making it possible to achieve long‐term and high‐throughput recording. To further minimize the initial damage induced by the surgery, we developed a surgical procedure that minimizes the electrode track to tens of micrometers (see Experimental Section, Figure 1c; Figure S3, Supporting Information). Figure 1d depicts the timeline of various procedures for examining the safety and effectiveness of the SHEA. Gait analyses were performed 1 day before and 1 week after implantation to evaluate the motor function, and immunostaining of SC tissue was performed for 2 weeks to 4 months after implantation to examine potential tissue damage and intraspinal recording quality.

### 2.2 Performance of SHEA Recording from SC Ventral Horn

**Figure 2:**
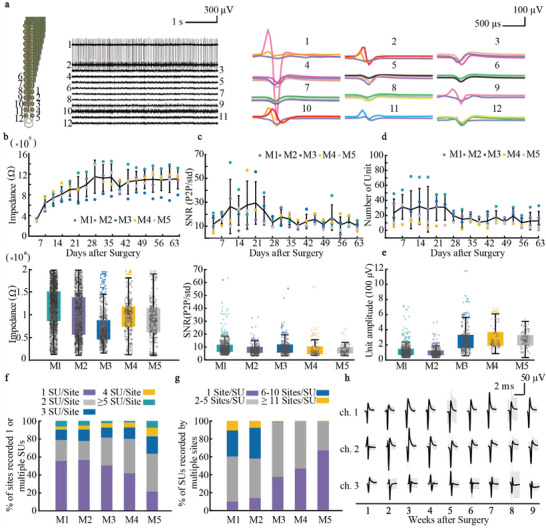
Electrophysiological recording performance of implanted SHEA. a) Typical raw data and sorted units from 12 recording sites. Left, a light microscopic image of one SHEA‐128 shank. Middle, raw data recorded from 12 sites marked on the left panel. Right, sorted units from 12 recording sites. b) Upper panel, impedance change over time. Colored dots represent the average impedance of functional channels in each recording session. The error bar indicates the standard deviation. Lower panel, the impedance distribution of each mouse. Black circles represent the impedance of each recording site in each recording session, and colored circles represent outliers. M1‐5: mouse 1–5. c) Upper panel, SNR change over time. Colored dots represent the average SNR of recorded units in each recording session. The error bar indicates the standard deviation. Lower panel, the SNR distribution of each mouse. Black circles represent the SNR of each unit in each recording session, and colored circles represent outliers. d) Number of units change over time. Colored dots represent the number of units of each recording session. The error bar indicates the standard deviation. e) Distribution of the amplitude of all sorted units for each mouse (*n* = 5 mice). Black circles represent the amplitude of each unit in each recording session, and colored circles represent the outliers. f) The density of recorded neurons per recording site for each mouse (*n* = 5 mice). SU: single unit. g) The number of recording sites that captured the same single units for each mouse (*n* = 5 mice). h) The waveform of units that was sorted from 3 typical recording sites within 9 contiguous weeks. The waveforms were extracted from 30 min recording segments. Error bars: standard deviation.

To examine the electrophysiological performance of the SHEA, we implanted SHEA into the SC ventral horn of 5 mice (M1‐M5), using either SHEA‐128 (M1, M2) or SHEA‐64 (M3‐M5), covering SC segments L1 to L2. The recording lasted for 9 weeks, including 18 recording sessions (twice per week). Intraspinal electrical signals, mostly single units, were detected within hours after electrode implantation, as shown by signals recorded at 12 example recording sites in one SHEA (**Figure** **2**a). The average impedance of the electrodes gradually increased and stabilized at 1 mω after 4 weeks in all mice (Figure 2b; Figure S4, Supporting Information). The signal‐to‐noise ratio (SNR) at most recording sites was relatively stable within the range of 10–30 throughout the 9 weeks (Figure 2c). In all 5 mice, the mean number of detected single units (SU) per recording session remained stable at ≈32 per electrode over the first 3 weeks after implantation and dropped to a lower level of ≈12 per probe during the subsequent 6 weeks (Figure 2d). The average amplitude of unit activities recorded in each mouse was between 89 to 260 µV (Figure 2e). The relative stability and consistency of the average SNR, unit yield, and unit amplitude over time demonstrated the SHEA's reliability for chronic SC recording.

The SHEA allowed the sampling of SUs by multiple recording sites, which were spaced at a distance lower than the detection range for SUs, resulting in a higher SU yield and improved accuracy of SU isolation. We showed SHEA‐128 and SHEA‐64′s SUs recording performance. Figure 1f demonstrates how many SUs could be captured by one recording site, and Figure 1g illustrates how many recording sites an SU could spread. In our experiment setup, we found that 91% of the SUs could be captured by electrode sites within 160 µm distance (≈12 recording sites of SHEA‐128 and ≈5 recording sets of SHEA‐64). With this multi‐sampling recording capability, we could determine that SUs accounted for 83 ± 13% (*n* = 5 mice) of all the units recorded by SHEA. More specifically, SHEA‐128 has a better performance (96 ± 3%) than SHEA‐64 (75 ± 10%) in identifying SUs (Figure S5, Supporting Information). Among all recording sessions, 55% (SHEA‐128, SHEA‐64) of SU‐capturing recording sites could capture two or more SUs. Such SU sampling by multiple electrodes could provide a robust long‐term recording of SUs for monitoring SC activity underlying motor control. This was demonstrated by the SUs recorded from three examples of SHEA recording sites over a period of 9 weeks (Figure 2h). It is worth mentioning that we can still collect intraspinal SUs one year after implantation (Figure S6, Supporting Information).

### 2.3 Effects of the SHEA Implantation on Immune Responses and Gait

**Figure 3:**
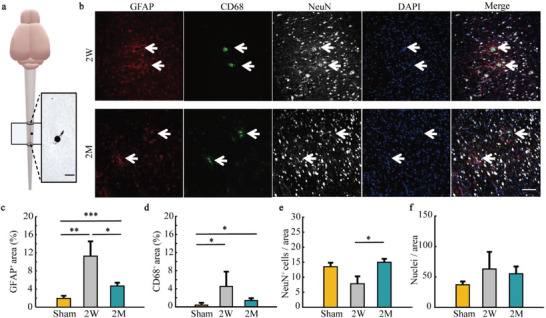
Chronic tissue responses of SC after SHEA implantation. a) The schematic of the spine implanted with SHEA. The electrode was implanted vertically in the ventral horn of the SC. The SC tissue was sliced perpendicular to the probe's insertion direction, and the magnified image shows a bright field image of a 10 µm SC slice with a probe segment embedded in it. Scale bar, 50 µm. SHEA: spinal cord hyperflexible electrode array; SC: spinal cord. b) Representative images of chronic tissue responses to SHEA at 2W and 2m after implantation. The samples were stained with GFAP (red), CD68 (green), and NeuN (gray). 2W: 2 weeks; 2M: 2 months; GFAP: glial fibrillary acidic protein; NeuN: neurons; DAPI: 4′, 6‐diamidino‐2‐phenylindole. White arrows show the location of implanted probes. Scale bar, 100 µm. c–f) Immunohistochemical analysis of GFAP, CD68, NeuN, and DAPI around the implant sites. Data represent the mean ± SD (*n* = 3–6 mice, Student's *t*‐test, *p < 0.05; **p < 0.01; ***p < 0.001; and ****p < 0.0001). SD: standard deviation.

To investigate SHEA's biocompatibility, we examined the density and morphology of astrocytes (marked by GFAP), microglia (marked by CD68), and neurons (marked by NeuN) on the cross‐section of the sliced SC tissue near the implantation site. (**Figure** **3**; Tables S2 and S3, Supporting Information, see Experimental Section). The percentage of GFAP^+^ and CD68^+^ in the 2 W group was significantly higher than the sham group, (*p* = 1.64 × 10^−4^, *p* = 0.014), whereas at 2 months, both percentages were significantly reduced compared to the 2 W group (*p* = 0.03, *p* = 0.18) while remaining higher than the sham (*p* = 4.02 × 10^−4^, *p* = 0.024). However, 4 months after implantation, the percentage of GFAP^+^ and CD68^+^ returned to the baseline level (*p* = 0.48, *p* = 0.067, Figure S7b,c, Supporting Information). The thickness of immunoreactive layer of GFAP^+^ and CD68^+^ was ≈15 µm 2 weeks after implantation and decreased to 5–10 µm over time (see Experimental Section, Figures S8 and S9, and Tables S4 and S5, Supporting Information). These results indicate that immune responses induced by probe insertion were alleviated and almost gone 2–4 months after the implantation, demonstrating SHEA can chronically coexist with SC tissue. Furthermore, neuron counts have no significant difference between sham and 2W or 2m groups (Figure 3e,f; Figure S7d, Supporting Information, *p* = 0.18, *p* = 0.38). In summary, we observed minimal tissue response and negligible neuronal degradation surrounding the SHEA after chronic implantation, which suggests SHEA's outstanding biocompatibility in the SC.

To further quantify the influence induced by the SHEA implantation on the behavioral level, we analyzed the gait performance of 5 mice for 4 times (1 day before, 1, 3, and 7 days after the surgery, Figure S10, Supporting Information). Stance width (*p* = 0.41), swing time (*p* = 0.24), stride time (*p* = 0.052), and gait symmetry (*p* = 0.51) showed no significant change over the time course of the experiment (Table S6, Supporting Information). Taken together, SHEA possesses advanced biocompatibility and safety as the chronic implantation showed minimal tissue response and negligible behavioral effect.

### 2.4 Movement Decoding of Intraspinal Signals

**Figure 4:**
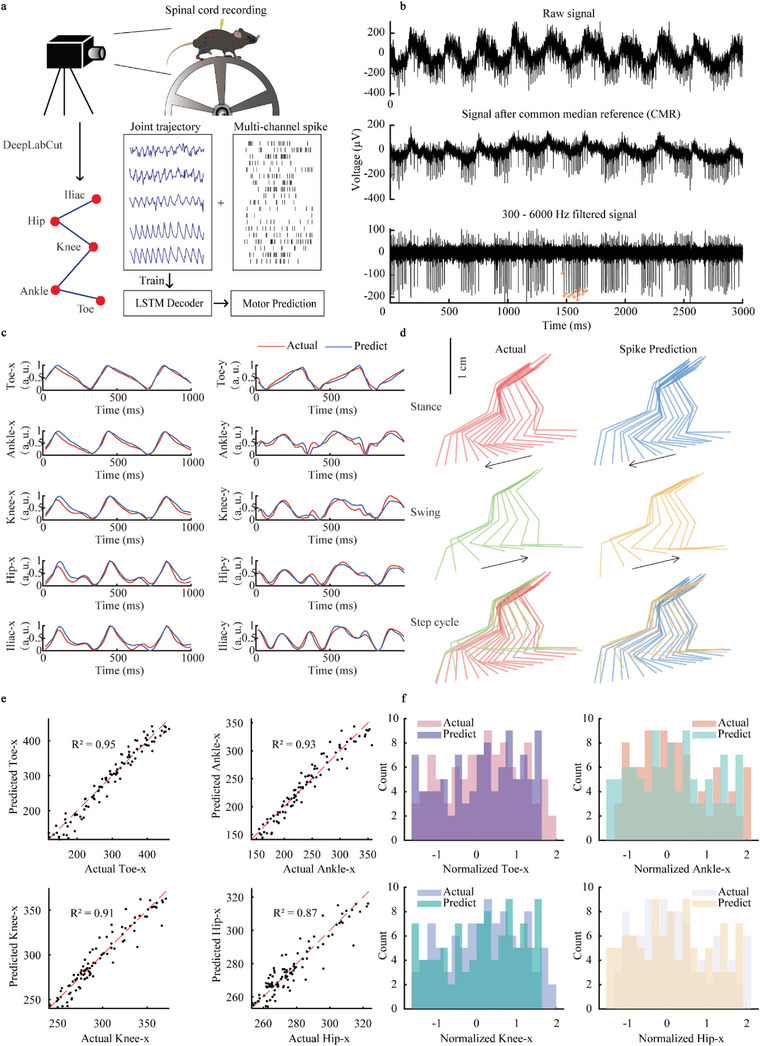
Decoding experiment setup and motor decoding results. a) The schematic outline of the decoding experimental procedure. Mouse hindlimb movements are captured in the camera and labeled as red dots in the schematic using DeepLabCut. An LSTM model is then trained using both behavioral and neural data to predict movements. b) The Top part displays a representative raw neural signal extracted from the spinal cord. In the center, the raw signal undergoes common median reference, with common mode noise removed. The lower portion shows the signal undergoing a bandpass filter (300‐6000 Hz), and orange stars mark the detected spikes’ time stamps. c) Actual (red) and decoded (blue) hindlimb joint coordinate traces. d) Original and spikes predicted hindlimb joint movement during a step cycle in 2D. e) Scatter plot comparison of actual and predicted results and the R^2^ values. Each data point represents the actual x‐coordinate value (*x*‐axis) paired with its corresponding predicted value (*y*‐axis) for toe, ankle, knee, and hip. The red dashed line is *x* = *y*. f) Histograms illustrating the counts of both actual and decoded x‐coordinates for toe, ankle, knee, and hip in a normalized range, showing a similar distribution between the two sets of data.

**Figure 5:**
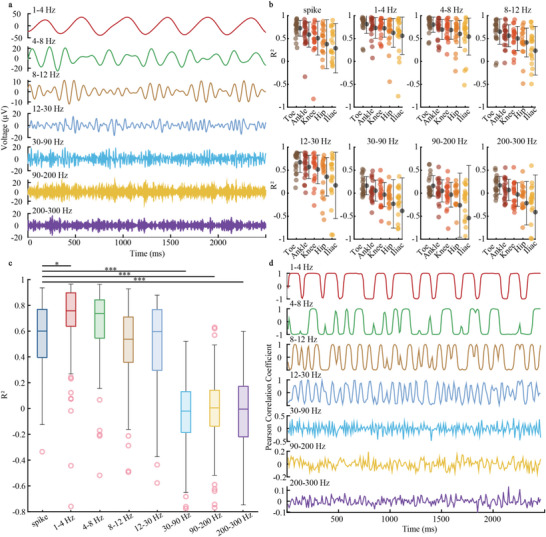
Overview of LFP data and decoding results comparison between spikes and LFP. a) Representative spinal cord LFP signals of seven frequency bands (1–4, 4–8, 8–12, 12–30, 30–90, 90–200, and 200–300 Hz) during movement. b) The decoding performance of each joint in mouse one using spikes and LFP with different bands. Colored dots represent the decoding R^2^ data points from each session. Black dots and error bars represent the average and standard deviation of R^2^. c) Comparison of decoding R^2^ of spike and LFP with different bands in mouse one; *p < 0.05, ***p < 0.0001. d) Pearson correlation coefficient between LFP with different bands and toe‐x coordinate during the running task.

SHEA's optimal biocompatibility and long‐term stability in the SC make it a promising tool for SC neural recording. Indeed, we showed the probe's outstanding performance in neural decoding by applying it to in vivo SC L3 ventral horn recording in mice during wheel‐running tasks and accurately decoded the motor behavior from the SC signals (**Figure** **4**a). We decoded hindlimb joints (toe, ankle, knee, hip, iliac) movement in two mice using spike and LFP (Figure 4b) with long short‐term memory (LSTM) neural network, which has been reported for its outstanding neural decoding performance.^[^ ^20^, ^21^ ^]^ We used the coefficient of determination (R^2^) as an evaluation metric for decoding results. We were able to accurately reconstruct the movement of hindlimb joints (Figure 4c,d; Figure S11, Supporting Information) with a decoding accuracy of up to 0.95 using spike signals (Figure 4e), and the decoded results share a similar distribution with the actual values (Figure 4f). We split the LFP into different bands (**Figure** **5**a) and checked their decoding performance on each joint. And we observed a difference in decoding performance between joints (Figure 5b; Figure S12, Supporting Information). We also compared the decoding performance of LFP and spike (Figure 5c, Figure S13, Supporting Information) and found that the R^2^ of 1–4 Hz LFP was comparable to or marginally better than the spike (*p* = 0.0432). However, spikes significantly outperformed LFP (Figure 5c, Figure S13, Supporting Information) in higher frequency bands (30–90 Hz, *p* < 0.0001; 90–200 Hz, *p* < 0.0001; and 200–300 Hz, *p* < 0.0001).

### 2.5 Neural Trajectory of Spike and LFP

**Figure 6:**
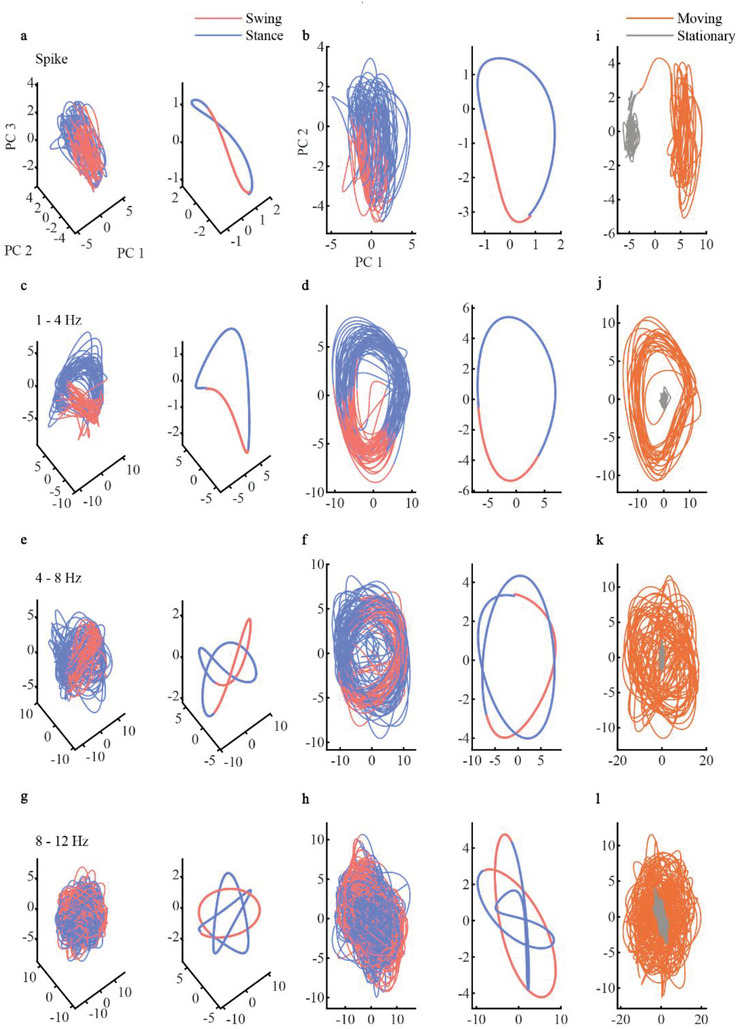
Neural trajectories of spinal cord spike and LFP during movement. a–h) Raw and average neural trajectories of spinal cord neural signals in 3D and 2D PCA space; red and blue lines represent a step cycle's swing and stance phases, respectively. All the Neural trajectories were recorded during movement. a) 3D Neural trajectory of spikes. b) 2D Neural trajectory of spikes. c) 3D Neural trajectory of LFP (1‐4 Hz). d) 2D Neural trajectory of LFP (1‐4 Hz). e) 3D Neural trajectory of LFP (4–8 Hz). f) 2D Neural trajectory of LFP (4–8 Hz). g) 3D Neural trajectory of LFP (8–12 Hz). h) 2D Neural trajectory of LFP (8–12 Hz). i–l) The neural trajectory for i) spike, j) LFP (1–4 Hz), k) LFP (4–8 Hz), and l) LFP (8–12 Hz) during stationary (gray lines) and moving (orange lines) stages.

To further explore the relationship between the SC signal and movement, we calculated the Pearson correlation between the LFP and the toe trajectory (Figure 5d). The results revealed that the lower‐frequency LFP, particularly in the range of 1–4 Hz, exhibited a strong correlation with the movement, whereas the higher‐frequency LFP did not. To gain another perspective, we visualized the neural trajectory using principal component analysis (PCA) in 3D and 2D.^[^ ^22^ ^]^ We observed periodic circle‐shaped spike trajectories (**Figure** **6**a,b), saddle‐shaped 1–4 Hz trajectories (Figure 6c,d; Figure S14, Supporting Information), knot‐shaped 4–8 Hz (Figure 6e,f), and 8–12 Hz trajectories (Figure 6g,h) during the swing (red lines) and stance (blue lines) phases of the step cycle; all of the neural dynamic rotated during step cycles repeatedly. And we also observed a clear neural state change of neural trajectory (Figure 6i–l) in the PCA space when the animal's state of motion switches between moving and stationary. However, we did not observe distinct geometry patterns in the neural trajectories of high‐frequency LFP (Figures S15 and S16, Supporting Information). Our findings suggest that during running, the SC neural dynamic of spikes and low‐frequency LFP exhibited specific geometry shape and periodic oscillations in low‐dimensional neural space, which agrees with the decoding results that spike and low‐frequency LFP in the ventral horn SC were more efficient in decoding hindlimb movement and the observation of the high correlation between low‐frequency LFP and movement.

## 3 Discussion

The mechanical mismatch between the conventional intraspinal electrodes and the SC tissue can cause electrode drift during the SC movement, influencing both short and long‐term recording stability. Furthermore, it can induce severe tissue response after implantation, eventually resulting in the loss of neural recording capability.^[^ ^23^, ^24^ ^]^ In this study, we developed the SHEA with an ultrathin structure conducted by advanced nanofabrication technologies. Owing to the hyper‐flexibility, SHEA experiences less torque and bending force than the conventional electrodes do when exposed to the same level of deformation caused by the SC movements, which prolongs the probe lifetime and contributes to its tissue affinity and chronic recording capability (Table S7, Supporting Information). As a result, SHEA achieved reliable chronic recording, accompanied by stable impedance,^[^ ^25^ ^]^ SNR, and detected unit yield events over 2 months. Additionally, the nanofabrication procedure also gives us high design flexibility to fit different intraspinal recording applications. It is also worth mentioning that reducing the electrodes’ thickness and width to enhance the flexibility and thereby improve the recording performance has been an emerging method in the development of more stable neural electrodes for brain applications.^[^ ^26^, ^27^, ^28^, ^29^ ^]^ However, due to the inverse anatomical structure of the spinal cord from the brain and its deformable and dynamic recording environment, the application of ultraflexible microelectrodes within the spinal cord has been very challenging in the past. This work represents a pioneering study to design and fabricate flexible neural electrodes to achieve chronic stable intraspinal recording.

Gait analysis indicated that SHEA exerted no behavior effect on stance width, stride length, swing time, and gait symmetry over 7 days after the implantation, and histology results showed that the immune response decreased significantly over time and led to a remarkable recovery to the baseline level 4 months after the implantation. Given that there is negligible chronic tissue response near the implantation location, we believe the initial implantation damage should be responsible for the immune responses. We also observed neuron counts increase from 2 weeks to 2 months after SHEA implantation, which may be caused by neuron regeneration or cell migration.^[^ ^30^, ^31^, ^32^ ^]^ Our findings demonstrated that SHEA induces minimal tissue response and negligible behavior effect over time after the implantation; therefore, we report that SHEA exhibited outstanding biocompatibility.

By fully utilizing the recording stability and the high resolution of the SHEA, the decoding results based on the intraspinal signal exhibit an accurate prediction of the hindlimb movements during the wheel running task. Compared to the cortical electrode, SHEA collects more specific motion‐related signals and shows less invasiveness.^[^ ^33^ ^]^ Previous studies have shown that intraspinal signals can be used to extract motor intent.^[^ ^34^, ^35^, ^36^ ^]^ Motor neurons in the ventral horn are involved in precise locomotor encoding compared to white matter and dorsal root ganglia. However, so far as we know, there are no electrodes that have been used to decode neurons in the ventral horn, which may be due to its low accessibility caused by its deep location.^[^ ^34^, ^37^ ^]^ In this study, we offered evidence showing a superior decoding result through motor neuron signals recorded from the ventral horn. Other than the neural signal itself, decoding performance is also affected by the labeling methods. The joint movement labeling accuracy can be enhanced by replacing our skin‐labeling technique with X‐ray tracking and 3D modeling.

In this experiment, we revealed a noteworthy observation: the lower‐frequency LFP, particularly in the range of 1–4 Hz, exhibited a strong correlation with the movement, whereas the higher‐frequency LFP did not. We observed that the hindlimb movement frequency was ≈3–4 Hz, close to the frequency range of 1–4 Hz in LFP. Interestingly, previous research in the motor cortex has reported similar findings, where low‐frequency LFPs were synchronized with movement direction,^[^ ^38^ ^]^ movement speed,^[^ ^39^ ^]^ and submovements.^[^ ^40^ ^]^ Since lower motor neurons are connected with the motor cortex, it is reasonable to expect similar phenomena in the SC. Therefore, we propose that the phase and frequency of the low‐frequency LFP in the SC could be synchronized with the movement output.

We demonstrated that flexible intraspinal electrodes could decode motor signals precisely by recording from the ventral horn, which directly captures motor neuron signals with minimal tissue damage. In this study, we also examined the accuracy of decoding performance with 16 (mean: 0.284, std: 0.910), 32 (mean: 0.4011, std: 0.573), and 64 (mean: 0.470, std: 0.502) channels. As the channel count increased, there was an upward trend in the mean R^2^ value; and that of 32 channels was close to the 64 channels group. However, we did not find statistical significance even though there was an increase in R^2^. This suggests that the decoding performance might be saturated at a moderate channel in the wheel running task. One of the important findings is that we could decode movement using SC spike and LFP, and we believe we can still perform accurate motor decoding for a long period after implantation, as LFP has been demonstrated as a stable and reliable signal source for neural decoding.^[^ ^41^, ^42^ ^]^ Moreover, spikes and moving averages of low‐frequency LFP, especially 1–4 and 4–8 Hz, achieved high decoding accuracy, consistent with previous results.^[^ ^43^ ^]^ We also observed distinct neural trajectories of spike and low‐frequency LFP during running in the SC for the first time in mammals. Interestingly, a similar phenomenon was recently reported in the spike signal from turtle SC,^[^ ^44^ ^]^ which may suggest that the control of rhythmic behavior in the SC may be evolutionarily conserved and naturally designed in a simple form to reduce energy consumption.^[^ ^45^, ^46^ ^]^ With the advantage of SHEA's chronic recording capability in the SC, we are interested in further exploring the mechanism of SC control of precise, non‐rhythmic behaviors such as target reaching and climbing. On the other hand, a growing body of evidence has demonstrated that intraspinal stimulation could restore motor and bladder function after spinal cord injury.^[^ ^47^, ^48^, ^49^ ^]^ However, making a stable spinal cord microstimulation electrode with minimal tissue invasiveness and negligible behavior effect is technically challenging. Our current results have shown that SHEA can survive tens of millions of stimulation pulses (45 µA, 300 Hz, cathodic charge‐balanced pulses), and SHEA can induce significant hindlimb displacement in anesthetized mice with only 1 µA intraspinal stimulation pulses. We believe that with SHEA's outstanding biocompatibility and its potential precise and stable stimulation performance, further clinical applications can be developed. We are also interested in exploring the potential of SHEA on SC injury animal models to monitor SC physiology during recovery and demonstrate the possibility of motor decoding using residual SC signals to control supporting devices for facilitating motor rehabilitation. Our recent results have shown that SHEA performed well as a neuromuscular stimulator. Since we were able to extract motion information from the recorded spike signal, the information may potentially guide us to optimize SC micro‐stimulation parameters to generate “correct” movement commands by mimicking or reproducing the spike signal under healthy conditions for more natural motor recovery.

## 4 Experimental Section

### The SHEA Fabrication and Preparation

Micro/nanofabrication technologies were employed to fabricate the SHEA, similar to the previous reports.^[^ ^26^, ^50^, ^51^ ^]^ The multilayer probes were fabricated using photolithography and electrical beam vaporization on a release layer deposited on a silicon wafer (300 nm SiO_2_, Rdmicro). The insulating layer was made of SU‐8 (SU‐8 2000.5, MicroChem Corp) or PI (PI2610, HD MicroSystem). Electrodes and interconnects that transmit intraspinal signals in between two insulating layers were made of titanium and gold. Detailed dimensions of the SHEA‐64 and the SHEA‐128 can be found in Table S1 (Supporting Information). After fabrication, a printed circuit board with one or two 64‐pin connectors was soldered to the contact pads on the Si substrate. SHEA was released using nickel etchant and attached to the tungsten needles with polyethylene glycol before implantation.

### Animal

Adult male C57BL/6J mice (7 weeks) were used for all of the animal studies performed. Mice were housed individually in the animal facility of the Institute of Neuroscience, Chinese Academy of Sciences (at a 12‐h light‐dark cycle at 23 °C, with food and water ad libitum). All experiments were approved by the Animal Care and Use Committee of the Center for Excellence in Brain Science and Intelligence Technology, Chinese Academy of Sciences, Shanghai, China. (NA‐061‐2021)

### SHEA Implantation Surgery

Mice were anesthetized using isoflurane (3% for induction and 1−1.5% for maintenance) in compressed air and were secured in a stereotactic apparatus. The skin and tissue above the implantation location were removed to expose the spine. A laminectomy was performed at the T11 or T12 vertebrae to expose the SC. The spine was immobilized with homemade spine holders, and the dura mater above the targeted SC was removed. A SHEA device with tungsten needles was mounted on a stereotaxic frame and inserted vertically into the SC. Once the probe reached the target location, saline water was applied to the attachment for a few minutes in order to dissolve polyethylene glycol, allowing tungsten needles to detach from the probe. Tungsten needles were then retracted from the tissue, leaving the flexible SHEA alone embedded in the SC. A coverslip with a size similar to the removed vertebrae was used to cover and thus protect the exposed SC, and the space between the coverslip and the surrounding vertebrae was filled with silicone elastomer. The wound was sealed by applying cyanoacrylate and followed up with dental cement (C&B‐Metabond, Parkell).

### Neural Recording

Intraspinal signals were collected twice a week, lasting at least 2 months post‐implantation for each mouse. An Intan RHD 2000 recording system with a 128‐channel headstage (Intan Technologies) was used to record neural signals. Homemade titanium spine holders were used as the reference. Signals were acquired with a 20 000‐sampling rate for 30 min in each recording session. The impedance of the electrode was measured using the same recording system under 1 kHz. Data were collected and processed from all the functional recording sites whose impedance was between 100 kΩ and 2 mω. All spike data were preprocessed in MATLAB (MathWorks) with customized scripts. A 300–6000 Hz bandpass filter and a 50 Hz notch filter were applied to the intraspinal raw signal. Spinal signals were collected from anesthetized mice with isoflurane (3% for induction; 1–1.5% through recording) and from behaving mice. To minimize the effect of the noise introduced during movements, a 50 ms moving window was applied to the time series collected from mice in the awake state to identify and remove any scope of data whose standard deviation was greater than 25 µV.^[^ ^52^ ^]^ The spike signal was then re‐referenced to their common median to adjust the baseline.^[^ ^26^ ^]^ MountainSort was utilized to transform the time series to unit activities generated by neurons.^[^ ^53^ ^]^ MountainSort re‐implemented the 300–6000 Hz bandpass filter and detected candidate action potentials by thresholding the raw signal amplitude. After spike sorting, yielded units were classified as single units if more than 1% of inter‐spike intervals were shorter than 3 ms.^[^ ^54^ ^]^

### Immunohistochemistry

Three mice were used in each immunoreaction assessment for the SHEA implantation. Mice were anesthetized with ethyl carbamate (0.3 mL, 15% w/v, Bioss, D10313), and the SC was perfused with cold PBS, followed by 4% paraformaldehyde. After removal, the implanted SC segment was post‐fixed overnight in 4% paraformaldehyde and gradient dehydrated with 10%, 20%, and 30% sucrose solution, respectively, at 4 °C. Tissue samples were then embedded in OCT (Leica, 14 020 108 926) and frozen overnight in a −80 °C freezer for cryosection. Horizontal cryostat sections (10 or 18 µm thick) were collected for immunohistochemical labeling. After rinsing in PBS, the sections were blocked with 3% normal goat serum in 0.5% T‐PBS (0.5% Triton‐X 100 in PBS) for an hour at room temperature. The sections were then incubated with the mixed primary antibodies: GFAP (1:900, Abcam, ab7260), CD68 (1:900, BIO‐RAD, MCA1957), and NeuN (1:900, Abcam, EPR12763) in antibody dilution buffer for an hour at room temperature. Secondary antibodies (all from Invitrogen) incubated sequentially for 2 h at room temperature were Alexa Fluor 488‐conjugated goat anti‐rat (1:2000, A‐48262); Alexa Fluor 594‐conjugated goat anti‐rabbit (1:2000, A‐11037) and Alexa Fluor 647‐conjugated goat anti‐mouse (1:2000, A‐21236). Before rinsing with PBS, the sections were stained with DAPI (Beyotime, C1002) for 2 min. SC sagittal sections were imaged with a confocal laser‐scanning microscope (Olympus FV3000).

### Immunohistochemical Analysis

The GFAP and CD68 staining of SC were collected and analyzed separately at 2 weeks, 2 months, and 4 months after SHEA implantation to visualize immunoreaction. Cell nuclei were stained by incubation with DAPI, as described above. In addition, the targeted SC sections were stained with antibodies against NeuN to quantify the neurons around the delivered SHEA. Images of 3–4 sections for each mouse (*n* = 3) were taken under a 20X objective, and 1–3 implantation sites for each section were selected for quantification. The interested areas centered on the implantation site with a diameter of ≈160 µm (minimum area containing all positive staining) in each group, and the positive area was measured using Image J software. The mean of the thickness around the implantation sites of GFAP and CD68 in eight directions (0°, 45°, 90°, 135°, 180°, 225°, 270°, and 315°) in each SC section was calculated. Regions away from implantation sites on SC were analyzed by the same method as sham groups. The data in this part were analyzed by ImageJ software and then plotted figures by MATLAB.

### Gait

Gait was measured on 5 mice at 4 different time points: 1 day before; and 1, 3, and 7 days after implantation surgery. Each mouse was allowed to run from one end of a transparent acrylic tunnel to the other end while being videotaped by a 100 fps camera for three trials. All videotapes were processed by the gait analysis software Visual Gait Lab to extract various variables pertaining to mice's gait performance.^[^ ^55^ ^]^ In brief, each videotape was first clipped so that only the footage where the mouse was running in a straight line with a stationary velocity was preserved. Visual Gait Lab, through a pre‐trained Convolutional Neural Network, identified the position of several body parts and their movement status, stance, or swing, throughout the clipped video on a frame‐by‐frame basis. Four variables, stance width, stride time, swing time, and gait symmetry, were extracted from the analyzed footage to assess mice's gait performance. A one‐way repeated multivariate analysis of variance was selected to evaluate the statistical significance of the impact of the implant on mice's gait.

### Motor Decoding Preparation

Two intact mice that showed voluntary wheel‐running behavior for further training were chosen. To motivate the mice to run continuously on the wheel, their water source was replaced with an unlimited 2% citric acid solution in their home cage. Once the mice were accustomed to running on the wheel, SHEA‐128 was implanted into the right ventral horn of the L3 spinal segment, with the electrode shanks parallel to the SC midline. The mice were allowed to recover after the surgery. Then, the hair over the ankle, knee, hip, and iliac joints was removed and marked with water‐soluble dye to improve the accuracy of joint tracking. An industrial camera (Hikvision, MV‐CA004‐10GC) at 60 fps was used to capture the hindlimb movement during the wheel running task. The SC signal was also recorded using the RHD 2000 (Intan Technology) recording system at 20 kHz and the timestamps were synchronized with the camera using a digital trigger. The *x* and *y* coordinates of the five hindlimb joints were extracted later using DeepLabCut.

### SC Neural Data Preprocessing

First, disconnected channels for each animal were removed. A common median reference was applied to the raw SC signal to reduce the overall motor noise across channels and enhance signal quality.^[^ ^56^ ^]^ Then, 300–6000 and 1–300 Hz 4th order butter worth zero‐phase bandpass filters were applied to the signal to extract high‐frequency spike signal and low‐frequency LFP. To extract spike trains from the high‐frequency signal, a thresholding method based on the mean and root mean square of the signal was applied. Timestamps that crossed *mean‐4.5rms* during the depolarizing phase as spikes for mice one and *mean‐3rms* for mice two, which had a lower amplitude level, were considered. As for the LFP signal, the same bandpass filter was used to extract local field potential bands in seven frequency ranges: 1–4, 4–8, 8–12, 12–30, 30–90, 90–200, and 200–300 Hz. Then the correlation between movement and LFP band was computed to gain a general perspective of the signal.^[^ ^57^ ^]^

### Motor Decoding Using SPIKE and LFP

For neural decoding, ten sessions of 1000‐frame behavior recording from each animal were used. Each frame was matched with a chunk of neural signal that occurred 300 ms before it. A 50 ms sliding window with a 10 ms time step was used to calculate the spike count for each chunk. As it was noticed that local motor potential exhibited promising motor decoding performance,^[^ ^43^ ^]^ and this concept was adapted by computing the moving average of each LFP frequency band using the same slide window method mentioned before. LSTM was used as the neural decoding method. The regression model consisted of two LSTM layers with layer normalization and dropout layers inserted between each LSTM layer. A 0.8:0.1:0.1 train‐valid‐test split was used for each training session and 60‐round Bayesian optimization was applied to find the optimal hyperparameters of the models. The optimization process was repeated 5 times for each session. The decoding performance was evaluated by calculating the coefficient of R^2^ of the outputs. Ten R^2^ values were obtained for joints from each session and averaged over 5 optimization repeats to get the result.

### PCA of Spinal Cord Neural Data

PCA was used to uncover the neural trajectories of the spike and each LFP band in low dimension relation to hindlimb movement. First, the signal recorded during movement was sorted and 62 units in mouse 1 and 40 units in mouse 2 were obtained. The firing rate of the spike trains was estimated with a 50 ms Gaussian kernel in MATLAB. Then PCA was applied on a time‐channel matrix to obtain the first three PCs of the spikes. For LFP, the data were downsampled to 1000 Hz and the same method was used to reduce the dimensionality. The total and average neural trajectories were visualized with the first three and two PCs. Red represented the swing phase and blue represented the stance phase of a step cycle. The neural trajectory of the spike and LFP during the state shift between stationary and moving was also visualized.

### Statistics

Data were presented as means ± SD. The results in the recording and the histology part using the Student *t*‐test were compared, as indicated in the figure legends. The stance width, stride time, swing time, and gait symmetry were compared using the one‐way repeated Multivariate Analysis of Variance test. Statistical evaluations of the decoding performance of spike and LFP (*n* = 8) were performed with one‐way ANOVA. In this study, statistical tests were performed using the SPSS software and MATLAB. A *p‐*value <0.05 was considered statistically significant.

## Conflict of Interest

The authors declare no conflict of interest.

## Author Contributions

J.F., X.‐C.L., and P.W. contributed equally to this work. X.L. conceived the project and designed the experiments. X.‐C.L. designed and fabricated the device. J.F., F.Y., and P.W. performed the animal experiments. P.W. developed and implemented decoding experiments and algorithms. J.F., P.W., F.Y., B.Z., and J.Y., analyzed the data and contributed to the figures. J.F., P.W., and F.Y. wrote the original manuscript. X.L. and Z.Z. reviewed and edited the manuscript. X.L. supervised the study.

## Supporting information

Supporting Information

## Data Availability

The data that support the findings of this study are available from the corresponding author upon reasonable request.

## References

[1] A. S. Buchman , D. A. Bennett , Expert Rev. Neurother. 2011, 11, 665. 21539487 10.1586/ern.11.57 PMC3121966

[2] G. B. D. M. N. D. Collaborators , Lancet Neurol. 2018, 17, 1083.30409709

[3] S. Harkema , Y. Gerasimenko , J. Hodes , J. Burdick , C. Angeli , Y. Chen , C. Ferreira , A. Willhite , E. Rejc , R. G. Grossman , V. R. Edgerton , Lancet 2011, 377, 1938. 21601270 10.1016/S0140-6736(11)60547-3 PMC3154251

[4] C. A. Angeli , M. Boakye , R. A. Morton , J. Vogt , K. Benton , Y. Chen , C. K. Ferreira , S. J. Harkema , N. Engl. J. Med. 2018, 379, 1244. 30247091 10.1056/NEJMoa1803588

[5] F. B. Wagner , J.‐B. Mignardot , C. G. Le Goff‐Mignardot , R. Demesmaeker , S. Komi , M. Capogrosso , A. Rowald , I. SeEz , M. Caban , E. Pirondini , M. Vat , L. A. Mccracken , R. Heimgartner , I. Fodor , A. Watrin , P. Seguin , E. Paoles , K. Van Den Keybus , G. Eberle , B. Schurch , E. Pralong , F. Becce , J. Prior , N. Buse , R. Buschman , E. Neufeld , N. Kuster , S. Carda , J. Von Zitzewitz , V. Delattre , et al., Nature 2018, 563, 65. 30382197 10.1038/s41586-018-0649-2

[6] C. Marquez‐Chin , M. R. Popovic , Biomed. Eng. Online 2020, 19, 34. 32448143 10.1186/s12938-020-00773-4 PMC7245767

[7] N. Greiner , B. Barra , G. Schiavone , H. Lorach , N. James , S. Conti , M. Kaeser , F. Fallegger , S. Borgognon , S. Lacour , J. Bloch , G. Courtine , M. Capogrosso , Nat. Commun. 2021, 12, 435. 33469022 10.1038/s41467-020-20703-1 PMC7815834

[8] A. Rowald , S. Komi , R. Demesmaeker , E. Baaklini , S. D. Hernandez‐Charpak , E. Paoles , H. Montanaro , A. Cassara , F. Becce , B. Lloyd , T. Newton , J. Ravier , N. Kinany , M. D'ercole , A. Paley , N. Hankov , C. Varescon , L. Mccracken , M. Vat , M. Caban , A. Watrin , C. Jacquet , L. Bole‐Feysot , C. Harte , H. Lorach , A. Galvez , M. Tschopp , N. Herrmann , M. Wacker , L. Geernaert , et al., Nat. Med. 2022, 28, 260. 35132264 10.1038/s41591-021-01663-5

[9] A. B. Ajiboye , F. R. Willett , D. R. Young , W. D. Memberg , B. A. Murphy , J. P. Miller , B. L. Walter , J. A. Sweet , H. A. Hoyen , M. W. Keith , P. H. Peckham , J. D. Simeral , J. P. Donoghue , L. R. Hochberg , R. F. Kirsch , Lancet 2017, 389, 1821. 28363483 10.1016/S0140-6736(17)30601-3 PMC5516547

[10] M. Capogrosso , T. Milekovic , D. Borton , F. Wagner , E. M. Moraud , J.‐B. Mignardot , N. Buse , J. Gandar , Q. Barraud , D. Xing , E. Rey , S. Duis , Y. Jianzhong , W. K. D. Ko , Q. Li , P. Detemple , T. Denison , S. Micera , E. Bezard , J. Bloch , G. Courtine , Nature 2016, 539, 284. 27830790 10.1038/nature20118 PMC5108412

[11] Z. J. Sperry , K. Na , S. S. Parizi , H. J. Chiel , J. Seymour , E. Yoon , T. M. Bruns , J. Neural Eng. 2018, 15, 036027. 29521279 10.1088/1741-2552/aab55f PMC5938739

[12] C. L. Kolarcik , C. A. Castro , A. Lesniak , A. J. Demetris , L. E. Fisher , R. A. Gaunt , D. J. Weber , X. T. Cui , J. Neural Eng. 2020, 17, 046012. 32434161 10.1088/1741-2552/ab94d7 PMC7768891

[13] Z. J. Sperry , K. Na , J. Jun , L. R. Madden , A. Socha , E. Yoon , J. P. Seymour , T. M. Bruns , J. Neural Eng. 2021, 18, 046005. 10.1088/1741-2552/abe398 PMC12906853 33545709

[14] E. J. Welle , J. E. Woods , A. A. Jiman , J. M. Richie , E. C. Bottorff , Z. Ouyang , J. P. Seymour , P. R. Patel , T. M. Bruns , C. A. Chestek , IEEE Trans. Neural Syst. Rehabil. Eng. 2021, 29, 993. 34014825 10.1109/TNSRE.2021.3082056 PMC8459724

[15] A. Prasad , Q.‐S. Xue , V. Sankar , T. Nishida , G. Shaw , W. J. Streit , J. C. Sanchez , J. Neural Eng. 2012, 9, 056015. 23010756 10.1088/1741-2560/9/5/056015

[16] N. W. Prins , R. Mylavarapu , A. M. Shoup , S. Debnath , A. Prasad , J. Neural Eng. 2020, 17, 016031. 31480029 10.1088/1741-2552/ab4104 PMC6960332

[17] R. L. Witkam , M. L. Buijse , I. J. J. Arnts , D. Henssen , K. C. P. Vissers , R. van Dongen , E. Kurt , Neuromodulation 2022, 25, 745. 35227584 10.1016/j.neurom.2022.01.012

[18] E. Cetinkaya , S. Gok , M. Sahin , Ann. Int. Conf. IEEE Eng. Med. Biol. Soc. 2018, 2018, 5069. 10.1109/EMBC.2018.8513408 30441480

[19] T. Kim , A. Branner , T. Gulati , S. F. Giszter , J. Neural Eng. 2013, 10, 045001. 23723128 10.1088/1741-2560/10/4/045001 PMC3723769

[20] J. I. Glaser , A. S. Benjamin , R. H. Chowdhury , M. G. Perich , L. E. Miller , K. P. Kording , eNeuro 2020, 7, 0506. 10.1523/ENEURO.0506-19.2020 PMC7470933 32737181

[21] G. K. Anumanchipalli , J. Chartier , E. F. Chang , Nature 2019, 568, 493. 31019317 10.1038/s41586-019-1119-1 PMC9714519

[22] J. P. Cunningham , B. M. Yu , Nat. Neurosci. 2014, 17, 1500. 25151264 10.1038/nn.3776 PMC4433019

[23] A. Ersen , S. Elkabes , D. S. Freedman , M. Sahin , J. Neural Eng. 2015, 12, 016019. 25605679 10.1088/1741-2560/12/1/016019 PMC4317289

[24] P. Fransen , Neuromodulation 2015, 18, 759. 25752652 10.1111/ner.12282

[25] J. P. Neto , P. Baião , G. Lopes , J. Frazão , J. Nogueira , E. Fortunato , P. Barquinha , A. R. Kampff , Front. Neurosci. 2018, 12, 715. 30349453 10.3389/fnins.2018.00715 PMC6188074

[26] L. Luan , X. Wei , Z. Zhao , J. J. Siegel , O. Potnis , C. A. Tuppen , S. Lin , S. Kazmi , R. A. Fowler , S. Holloway , A. K. Dunn , R. A. Chitwood , C. Xie , Sci. Adv. 2017, 3, e1601966. 28246640 10.1126/sciadv.1601966 PMC5310823

[27] J. E. Chung , H. R. Joo , J. L. Fan , D. F. Liu , A. H. Barnett , S. Chen , C. Geaghan‐Breiner , M. P. Karlsson , M. Karlsson , K. Y. Lee , H. Liang , J. F. Magland , J. A. Pebbles , A. C. Tooker , L. F. Greengard , V. M. Tolosa , L. M. Frank , Neuron 2019, 101, 21. 30502044 10.1016/j.neuron.2018.11.002 PMC6326834

[28] Z. Zhao , H. Zhu , X. Li , L. Sun , F. He , J. E. Chung , D. F. Liu , L. Frank , L. Luan , C. Xie , Nat. Biomed. Eng. 2023, 7, 520. 36192597 10.1038/s41551-022-00941-y PMC10067539

[29] T.‐M. Fu , G. Hong , T. Zhou , T. G. Schuhmann , R. D. Viveros , C. M. Lieber , Nat. Methods 2016, 13, 875. 27571550 10.1038/nmeth.3969

[30] F. M. Bareyre , M. Kerschensteiner , O. Raineteau , T. C. Mettenleiter , O. Weinmann , M. E. Schwab , Nat. Neurosci. 2004, 7, 269. 14966523 10.1038/nn1195

[31] G. Courtine , B. Song , R. R. Roy , H. Zhong , J. E. Herrmann , Y. Ao , J. Qi , V. R. Edgerton , M. V. Sofroniew , Nat. Med. 2008, 14, 69. 18157143 10.1038/nm1682 PMC2916740

[32] C. S. Ahuja , S. Nori , L. Tetreault , J. Wilson , B. Kwon , J. Harrop , D. Choi , M. G. Fehlings , Neurosurgery 2017, 80, S9. 28350947 10.1093/neuros/nyw080

[33] W.‐K. Tam , T. Wu , Q. Zhao , E. Keefer , Z. Yang , BMC Biomed. Eng. 2019, 1, 22. 32903354 10.1186/s42490-019-0022-z PMC7422484

[34] A. Prasad , M. Sahin , J. Neuroeng. Rehabil. 2012, 9, 41. 22713735 10.1186/1743-0003-9-41 PMC3443439

[35] Y. Fathi , A. Erfanian , Front. Neurosci. 2022, 16, 801818. 35401098 10.3389/fnins.2022.801818 PMC8990134

[36] D. J. Weber , R. B. Stein , D. G. Everaert , A. Prochazka , IEEE Trans. Neural Syst. Rehabil. Eng. 2006, 14, 240. 16792303 10.1109/TNSRE.2006.875575

[37] Y. Fathi , A. Erfanian , J. Neural Eng. 2021, 18, 026015. 10.1088/1741-2552/abd82a 33395669

[38] J. Rickert , S. C. De Oliveira , E. Vaadia , A. Aertsen , S. Rotter , C. Mehring , J. Neurosci. 2005, 25, 8815. 16192371 10.1523/JNEUROSCI.0816-05.2005 PMC6725584

[39] S. Perel , P. T. Sadtler , J. M. Godlove , S. I. Ryu , W. Wang , A. P. Batista , S. M. Chase , Ann. Int. Conf. IEEE Eng. Med. Biol. Soc. 2013, 2013, 299. 10.1109/EMBC.2013.6609496 PMC4433020 24109683

[40] T. M. Hall , F. De Carvalho , A. Jackson , Neuron 2014, 83, 1185. 25132467 10.1016/j.neuron.2014.07.022 PMC4157580

[41] A. Jackson , T. M. Hall , IEEE Trans. Neural Syst. Rehabil. Eng. 2017, 25, 1705. 28113942 10.1109/TNSRE.2016.2612001 PMC6051483

[42] D. Wang , Q. Zhang , Y. Li , Y. Wang , J. Zhu , S. Zhang , X. Zheng , J. Neural Eng. 2014, 11, 036009. 24809544 10.1088/1741-2560/11/3/036009

[43] C. Gallego‐Carracedo , M. G. Perich , R. H. Chowdhury , L. E. Miller , J. A. Gallego , Elife 2022, 11, e73155. 35968845 10.7554/eLife.73155 PMC9470163

[44] H. Lindén , P. C. Petersen , M. Vestergaard , R. W. Berg , Nature 2022, 610, 526. 36224394 10.1038/s41586-022-05293-w

[45] J. E. Niven , S. B. Laughlin , J. Exp. Biol. 2008, 211, 1792. 18490395 10.1242/jeb.017574

[46] J. E. Niven , Curr. Opin. Neurobiol. 2016, 41, 129. 27664945 10.1016/j.conb.2016.09.004

[47] S. E. Mondello , M. R. Kasten , P. J. Horner , C. T. Moritz , Front. Neurosci. 2014, 8, 21. 24578680 10.3389/fnins.2014.00021 PMC3936503

[48] P. J. Grahn , K. H. Lee , A. Kasasbeh , G. W. Mallory , J. T. Hachmann , J. R. Dube , C. J. Kimble , D. A. Lobel , A. Bieber , J. H. Jeong , K. E. Bennet , J. L. Lujan , J. Neurosurg. 2015, 123, 232. 25479124 10.3171/2014.10.JNS132370 PMC4457704

[49] J. T. Hachmann , J. H. Jeong , P. J. Grahn , G. W. Mallory , L. Q. Evertz , A. J. Bieber , D. A. Lobel , K. E. Bennet , K. H. Lee , J. L. Lujan , PLoS One 2013, 8, e81443. 24339929 10.1371/journal.pone.0081443 PMC3855281

[50] X. Wei , L. Luan , Z. Zhao , X. Li , H. Zhu , O. Potnis , C. Xie , Adv. Sci. 2018, 5, 1700625. 10.1002/advs.201700625 PMC6010728 29938162

[51] Z. Zhao , X. Li , F. He , X. Wei , S. Lin , C. Xie , J. Neural Eng. 2019, 16, 035001. 30736013 10.1088/1741-2552/ab05b6 PMC6506360

[52] J. E. Chung , J. F. Magland , A. H. Barnett , V. M. Tolosa , A. C. Tooker , K. Y. Lee , K. G. Shah , S. H. Felix , L. M. Frank , L. F. Greengard , Neuron 2017, 95, 1381. 28910621 10.1016/j.neuron.2017.08.030 PMC5743236

[53] A. Tankus , Y. Yeshurun , I. Fried , J. Neural Eng. 2009, 6, 056001. 19667458 10.1088/1741-2560/6/5/056001 PMC2837589

[54] R. Mukamel , A. D. Ekstrom , J. Kaplan , M. Iacoboni , I. Fried , Curr. Biol. 2010, 20, 750. 20381353 10.1016/j.cub.2010.02.045 PMC2904852

[55] R. Fiker , L. H. Kim , L. A. Molina , T. Chomiak , P. J. Whelan , J. Neurosci. Methods 2020, 341, 108775. 32428621 10.1016/j.jneumeth.2020.108775

[56] J. D. Rolston , R. E. Gross , S. M. Potter , Ann. Int. Conf. IEEE Eng. Med. Biol. Soc. 2009, 2009, 1604. 10.1109/IEMBS.2009.5333230 19964004

[57] P. Berens , Front. Neurosci. 2008, 2, 199. 19225593 10.3389/neuro.01.037.2008 PMC2622750

