# Supplementary material for: A Hyperflexible Electrode Array for Long‐Term Recording and Decoding of Intraspinal Neuronal Activity
Source: Adv Sci (Weinh). 2023 Oct 23;10(33):2303377. doi: 10.1002/advs.202303377 (PMC10667843; doi:10.1002/advs.202303377)
Supplement: Supplementary file 1 — Supporting Information [file ADVS-10-2303377-s001.pdf]

ADVANCED SCIENCE
Open Access

Supporting Information

for *Adv. Sci.*, DOI 10.1002/advs.202303377

A Hyperflexible Electrode Array for Long-Term Recording and Decoding of Intraspinal Neuronal Activity

*Jie Fan, Xiaocheng Li, Peiyu Wang, Fan Yang, Bingzhen Zhao, Jianing Yang, Zhengtuo Zhao and Xue Li**

Supporting Information

A Hyperflexible Electrode Array for Long-term Recording and Decoding of Intraspinal Neuronal Activity

*Jie Fan, Xiaocheng Li, Peiyu Wang, Fan Yang, Bingzhen Zhao, Jianing Yang, Zhengtuo Zhao, Xue Li**

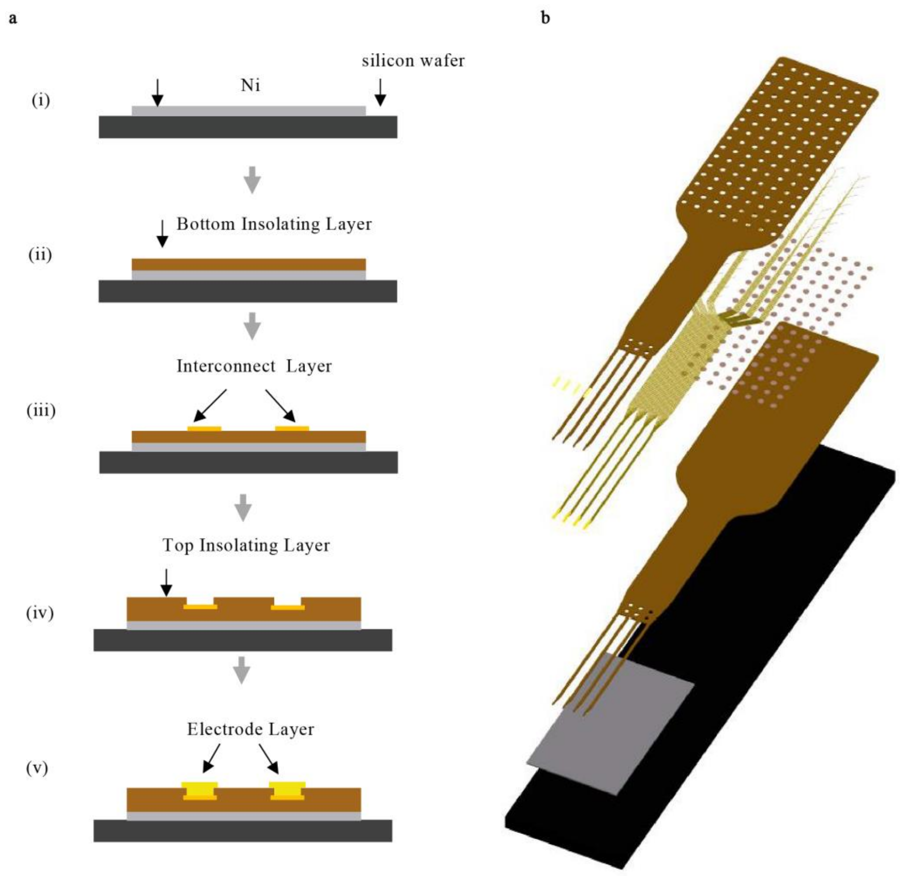

Supplementary Figure 1. The SHEA fabrication and layout. a, Schematic of the fabrication process for SHEA. b, Schematics showing layered SHEA structure from the electrode sites (topmost structure) to the silicon wafer (bottommost structure).

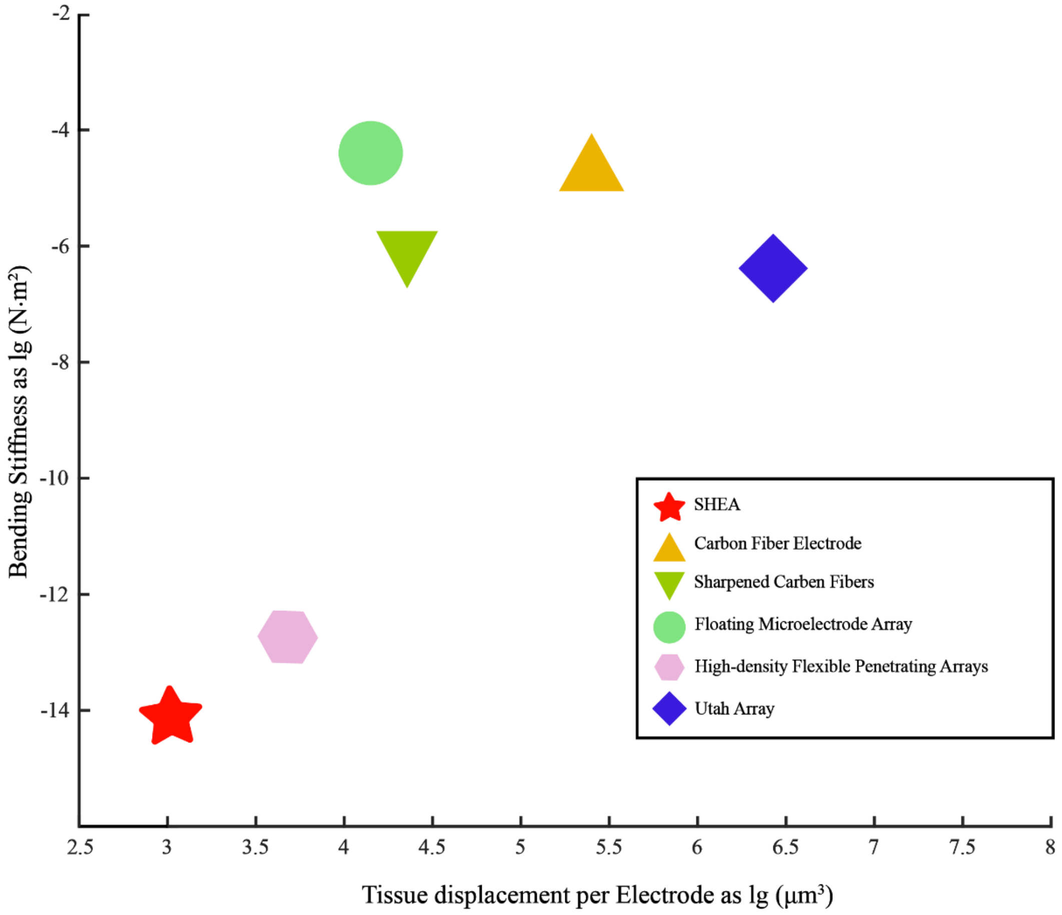

Supplementary Figure 2. Comparison of tissue displacement and bending stiffness of different intraspinal electrodes. Comparisons in bending stiffness (y-axis) and tissue displacement per electrode (x-axis) among different types of intraspinal SC electrodes. Carbon Fiber Electrode (Denes Budai and Z. Molnár, 2001), Sharpened Carben Fibers (Elissa J. Welle et al., 2021), Floating Microelectrode Array (Noeline W Prins et al., 2020), High-density flexible penetrating arrays (Zachariah J Sperry et al., 2021), Utah Array (Tim M Bruns et al., 2011).

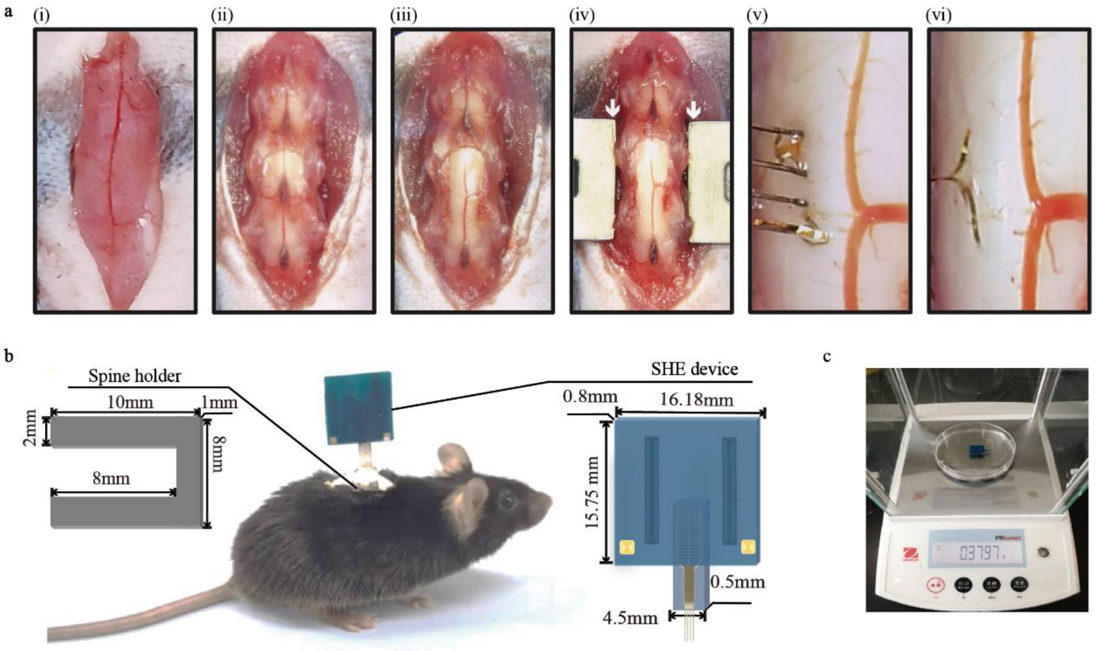

Supplementary Figure 3. Operation flow chart of the SHEA device implantation. a, Representative images of the SHEA implantation surgical procedure. (i) The exposed tissue after removing skin above the spine. (ii) The SC after retracting muscles and removing tissues overlying targeted vertebrae. (iii) The exposed spinal cord after laminectomy. (iv) The spine was fixed using a pair of spine holders marked by white arrows. (v) Electrodes and tungsten assembly were implanted in tissue after the epidural was retracted. (vi) Electrodes left in SC tissue after the tungsten needle was removed. b, A picture shows a mouse implanted with a SHEA device. Zoomed in view shows the schematic of the spine holder and the backend connection with dimensions. c, Total weight of the SHEA device (0.3797 g).

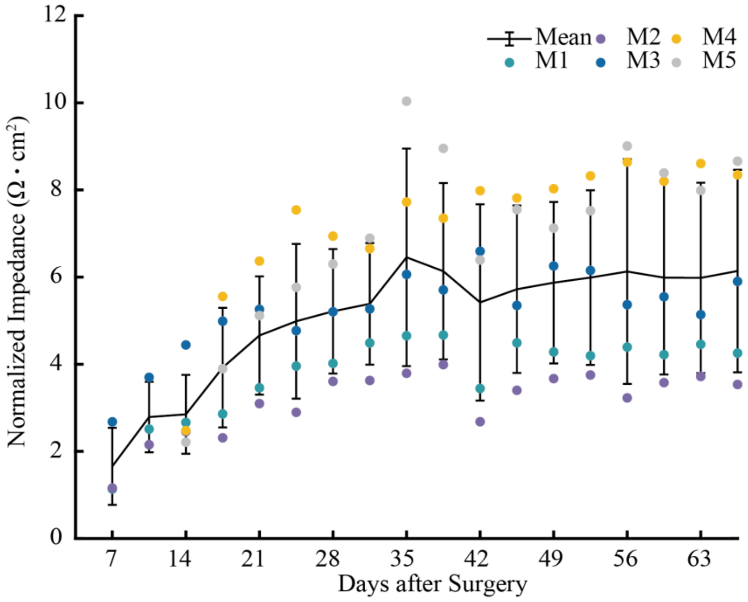

Supplementary Figure 4. The normalized impedance values (Ω·cm^2) of SHEA. Normalized impedance change over time. Colored dots represent the average impedance of functional channels in each recording session. The error bar indicates the standard deviation.

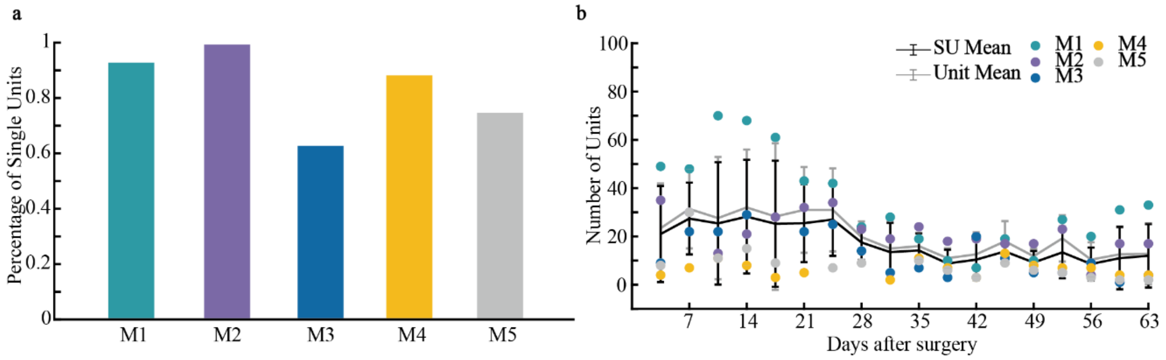

Supplementary Figure 5. Comparison between single units and total units. a, The percentage of single units out of all the units recorded from each mouse. b, The number of single units for each mouse on each recording session over time with the average number of single units (black line) and total units (gray line). The error bar indicates the standard deviation. M1-5: mouse 1-5.

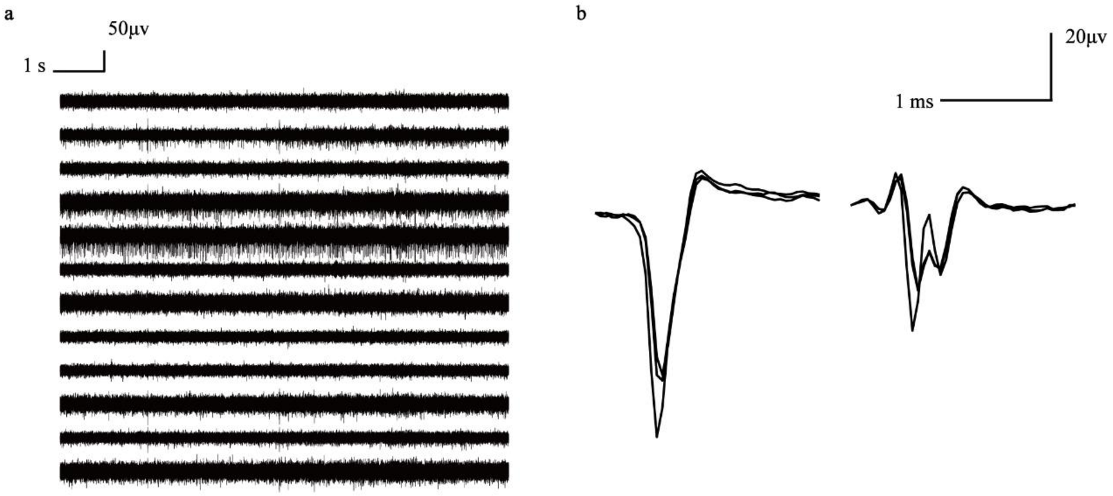

Supplementary Figure 6. Electrophysiological recording performance of SHEA one year after implantation. a, A segment of typical raw data and sorted units from 10 electrode sites in mouse 2 under anesthesia around one year after implantation. b, The waveforms of the unit sorted from mouse 2 under anesthesia around one year after SHEA implantation.

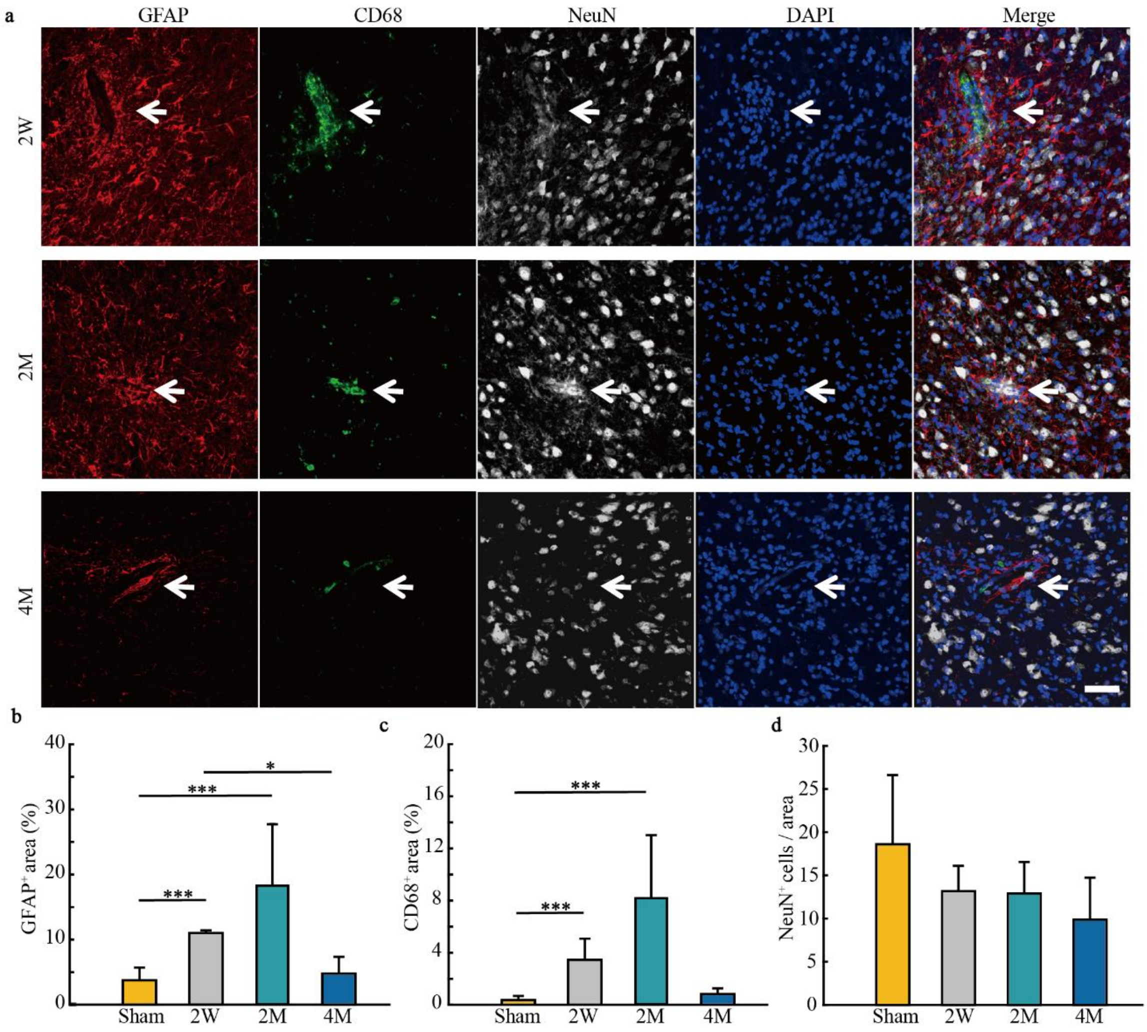

Supplementary Figure 7. Chronic tissue responses of SC after SHEA-128 implantation.
a, Representative images of chronic tissue responses to SHEA-128 at 2W, 2M, and 4M after implantation. 2W: 2 weeks; 2M: 2 months; 4M: 4 months; GFAP: glial fibrillary acidic protein; NeuN: neurons; DAPI: 4', 6-diamidino-2-phenylindole. White arrows show the location of implanted probes. Scale bar, 50µm. b-d, Immunohistochemical analysis of b, GFAP c, CD68 and d, NeuN around the implantation sites. Data represent the mean ± SD (*n*=3-6 mice, Student's t-test, * $p < 0.05$; *** $p <$ 0.001. SD: standard deviation.

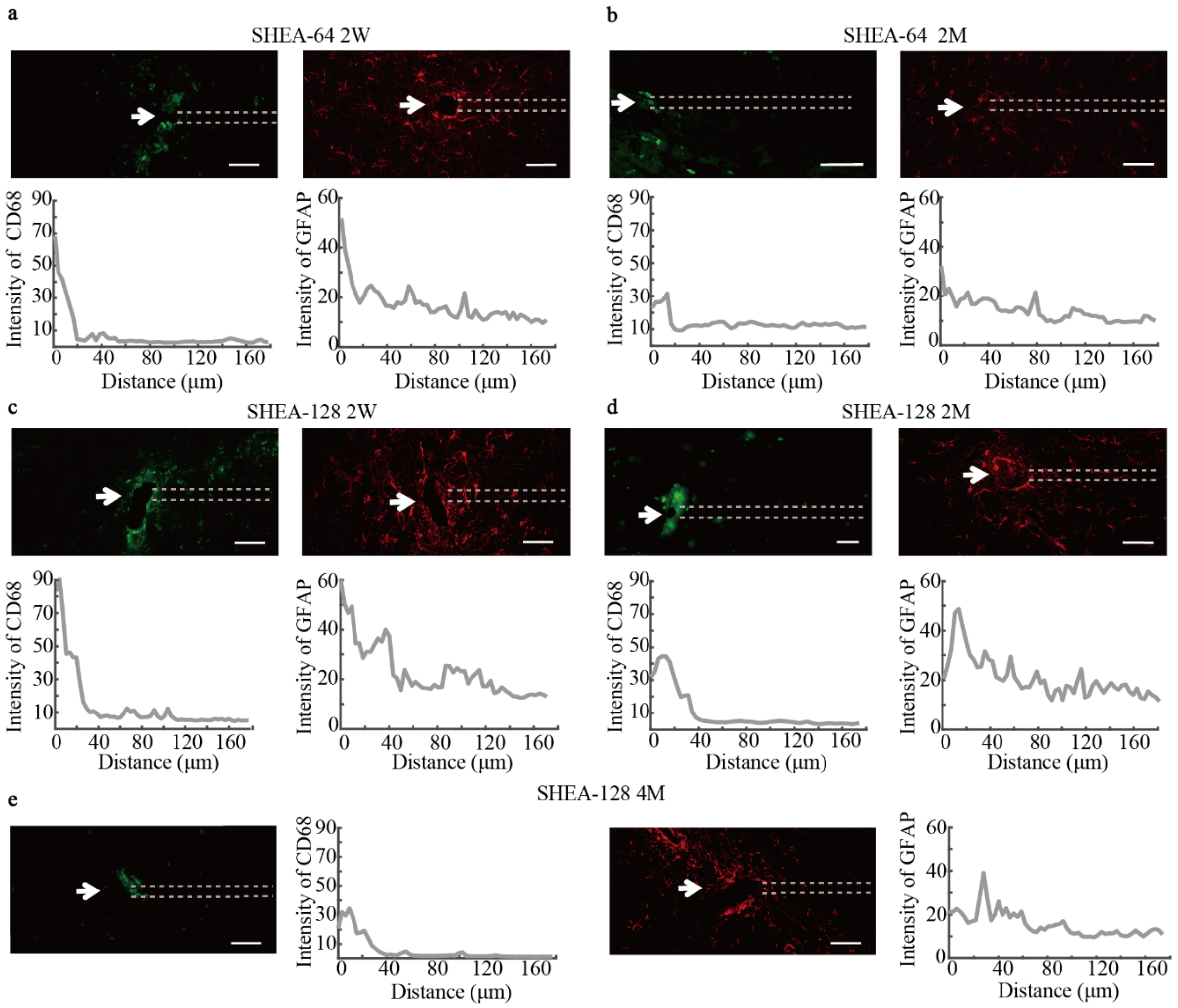

Supplementary Figure 8. Analysis of the fluorescence intensity span with the distance from the SHEA implantation site. Chronic tissue responses of SHEA-64 at a, 2W and b, 2M and SHEA-128 at c, 2W, d, 2M and e, 4M after implantation and their immunohistochemical analysis of CD68 (green) and GFAP (red). The Gray dotted line shows the analyzed region. White arrows show the location of implanted probes. All scale bars, 50 μm. 2W: 2 weeks; 2M: 2 months; 4M: 4 months; GFAP: glial fibrillary acidic protein.

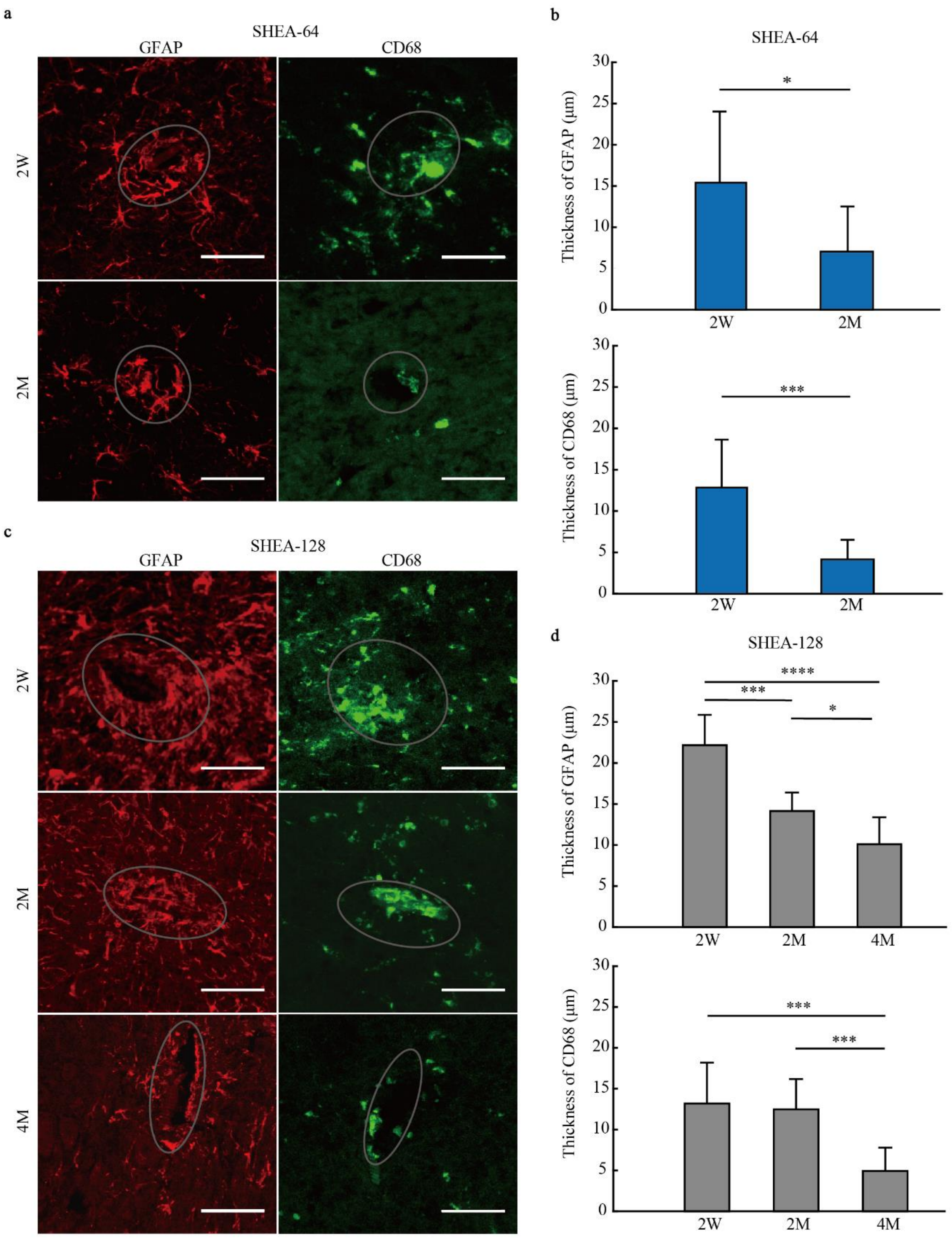

Supplementary Figure 9. Analysis of the immunoreactive layer thickness around the SHEA implantation site. a, c, Representative images of the immunoreactive around the a, SHEA-64, and c, SHEA-128 probe implantation sites. The gray circle indicates the analyzed area of the immunoreactive layers around the implantation sites. All scale bars, 50μm. b, d, The quantitative analysis of GFAP and CD68's thickness from 2W, 2M, and 4M groups. n = 3 mice. Data were presented as mean ± SD and were analyzed by Student's t-test. * $p < 0.05$; *** $p < 0.001$. 2W: 2 weeks; 2M: 2 months; 4M: 4 months; GFAP: glial fibrillary acidic protein.

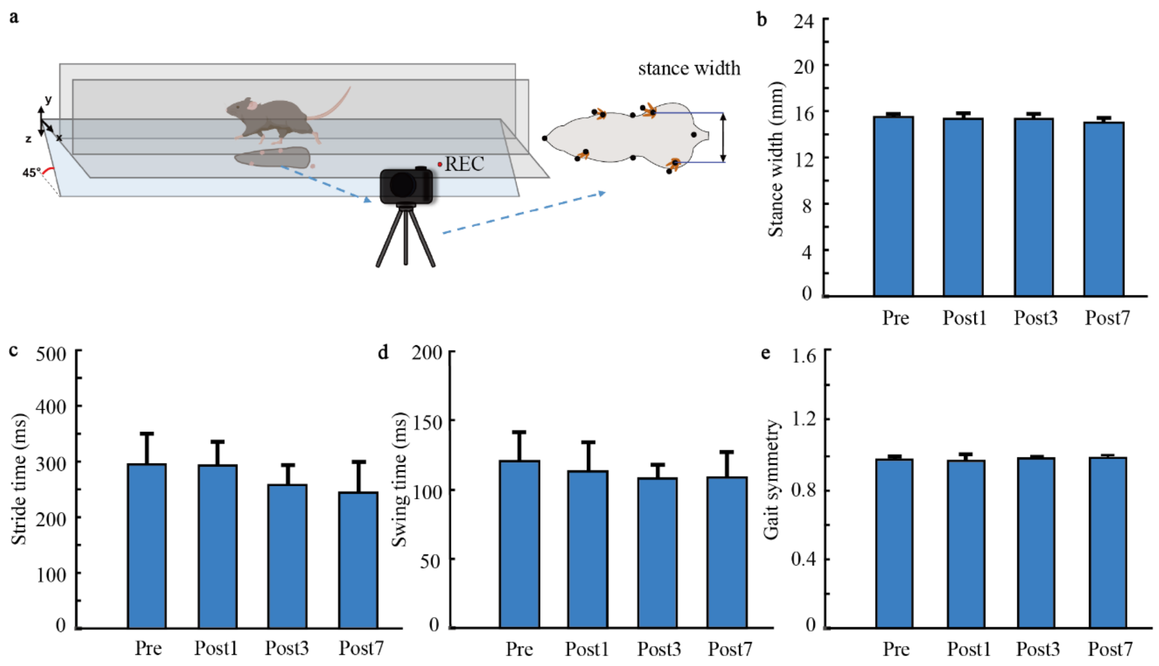

Supplementary Figure 10. Gait performance before and after the SHEA implantation. a, Gait experimental setup schematic. The black dots show the body parts identified by Visual Gait Lab as it tracked the mice’s movements. The change of: b, Stance width, c, Stride time, d, Swing time, and e, Gait symmetry before implantation and 1, 3, and 7 days post implantation. Data represent the mean ± SD (One-way repeated MANOVA, *n* = 5 mice). SD: standard deviation.

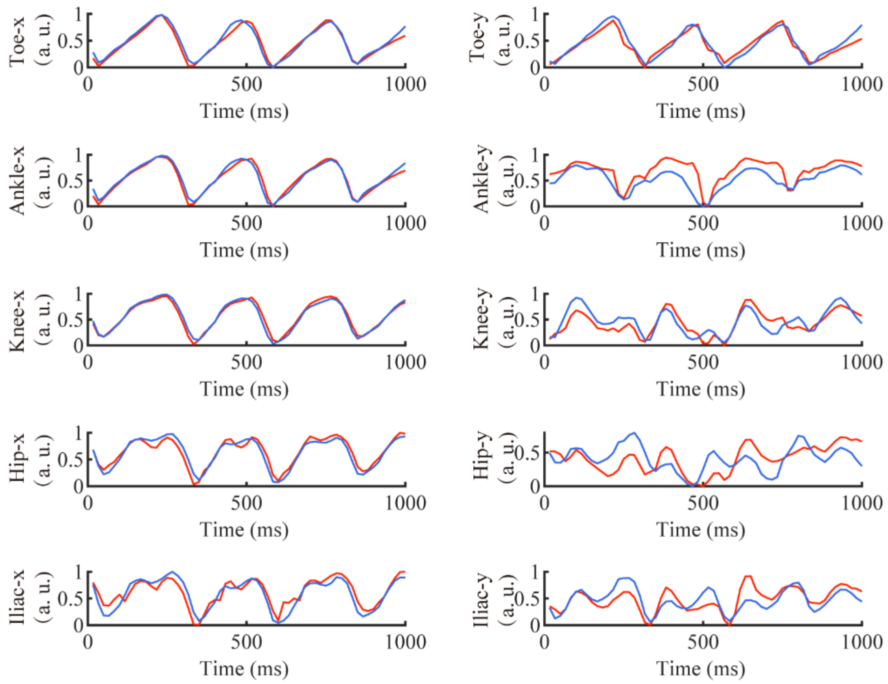

Supplementary Figure 11. Representative actual and decoded hindlimb joint coordinates trace in mouse 2. The red line represents the actual hindlimb joint coordinate traces. The blue line represents the decoded hindlimb joint coordinate traces.

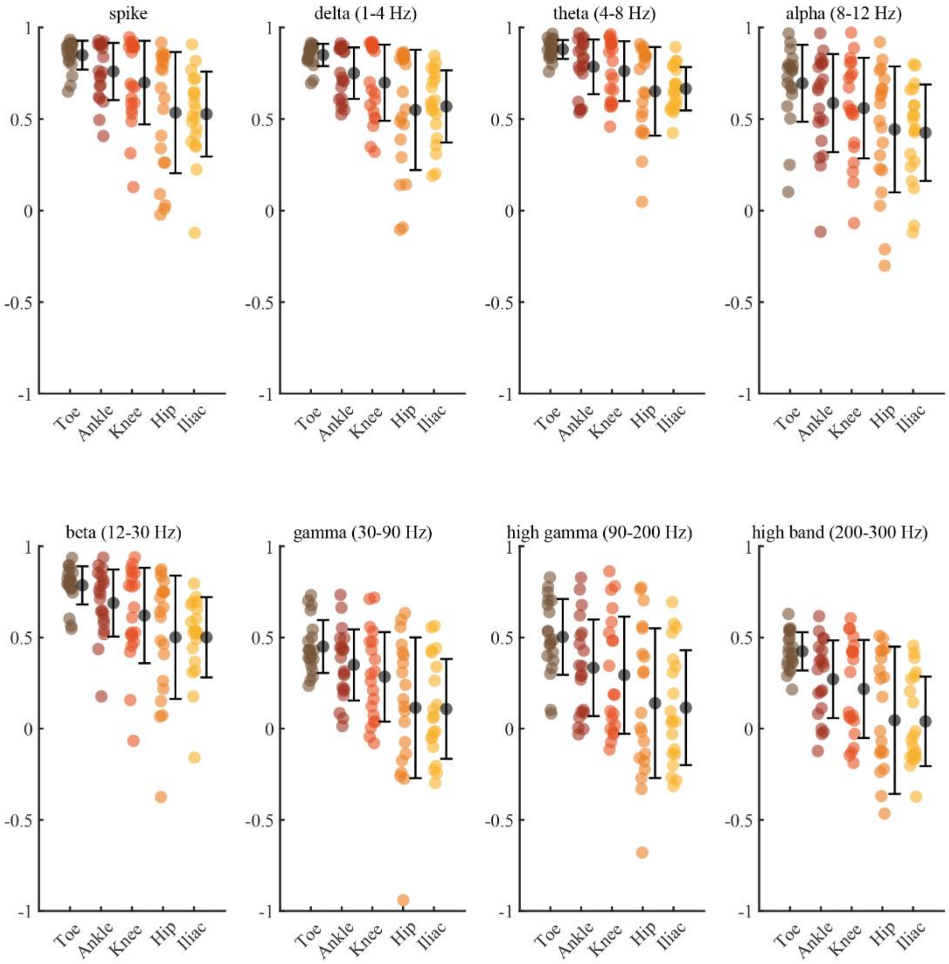

Supplementary Figure 12. Decoding performance (R^2) of spike and LFP of the position of the toe, ankle, knee, hip, and iliac joints in mouse 2. Colored dots represent decoding R^2 data points and error bars represent standard deviation.

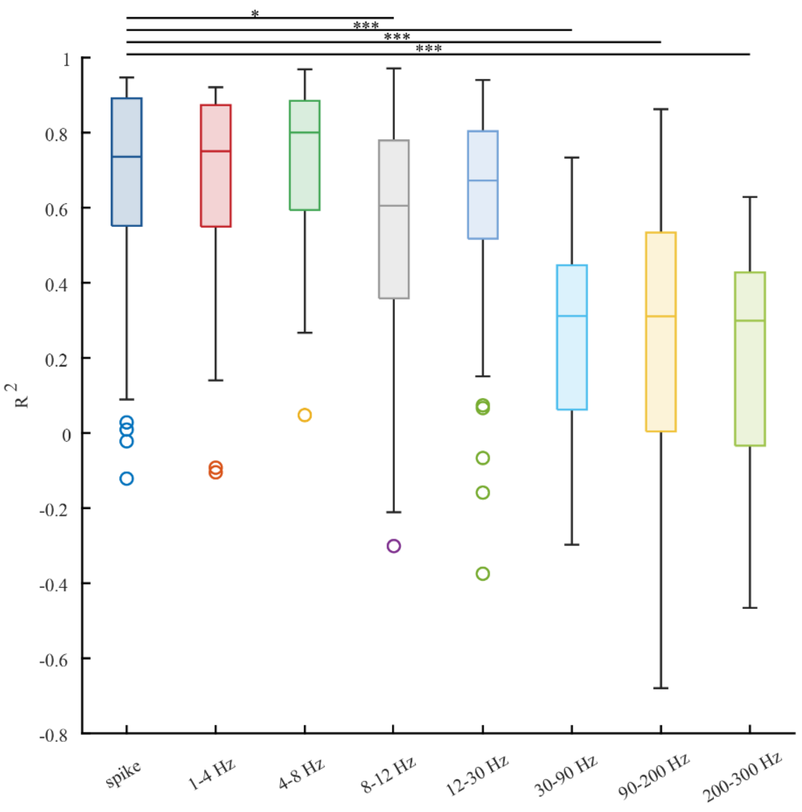

Supplementary Figure 13. Decoding R^2 of mouse 2. Comparison of decoding R^2 of different signal type in mouse 2; * $p < 0.05$, *** $p < 0.0001$. Error bars represent standard deviation, and colored circles represent outliers.

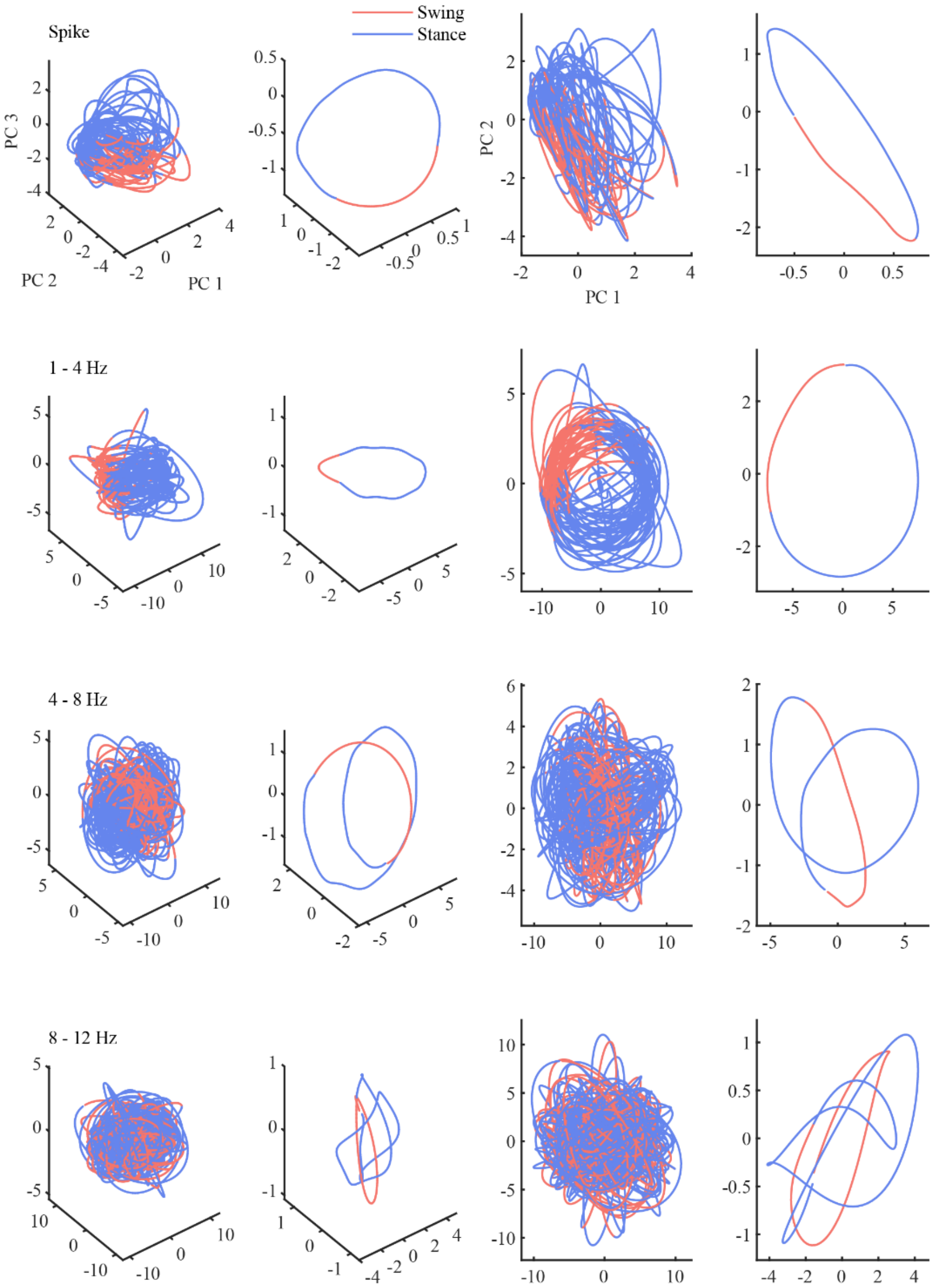

Supplementary Figure 14. Mouse 2 spike and low-frequency LFP neural trajectories. Raw and average neural trajectories in 3D and 2D PCA space of the spike (row 1), 1-4 Hz (row 2) ,4-8 Hz (row 3), and 8-12 Hz (row 4) LFP of mouse 2. Red and blue lines represent the swing and stance phases of a step cycle, respectively.

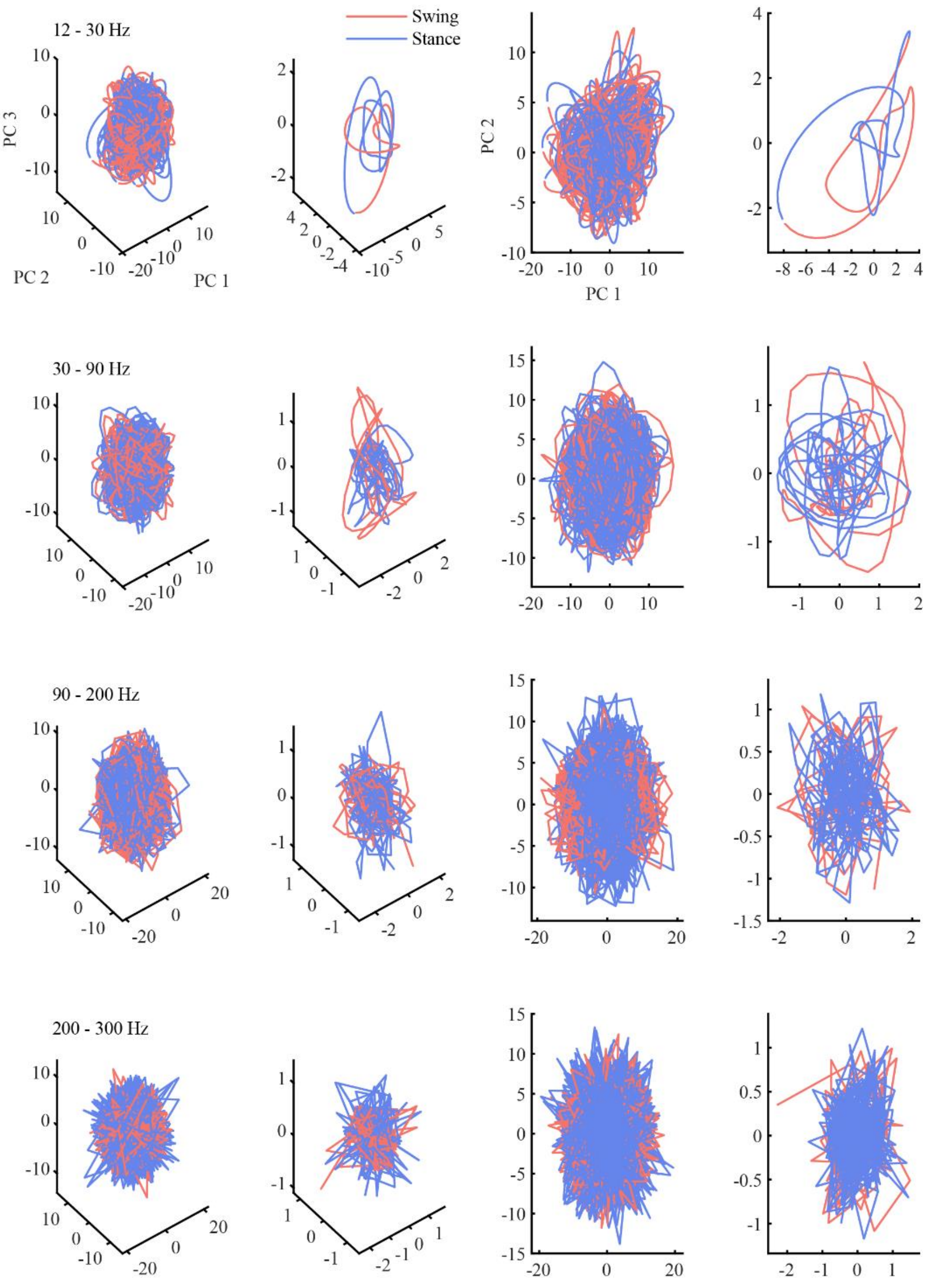

Supplementary Figure 15. Mouse 1 high-frequency LFP neural trajectories. Raw and average neural trajectories in 3D and 2D PCA space of the 12-30 Hz (row 1), 30-90 Hz (row 2) ,90-200 Hz (row 3), and 200-300 Hz (row 4) LFP of mouse 1. Red and blue lines represent the swing and stance phases of a step cycle, respectively.

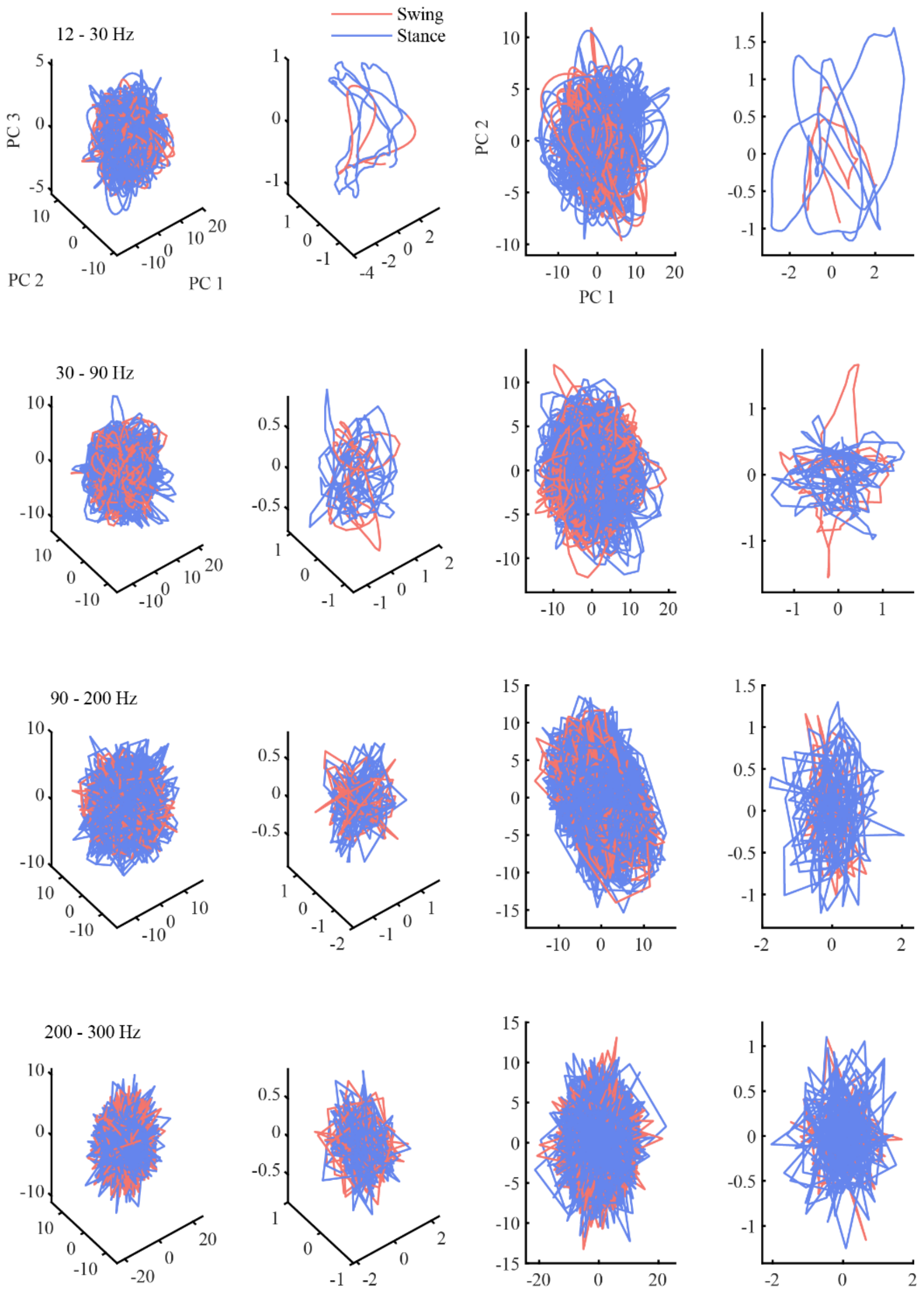

Supplementary Figure 16. Mouse 2 high-frequency LFP neural trajectories. Raw and average neural trajectories in 3D and 2D PCA space of the 12-30 Hz (row 1), 30-90 Hz (row 2) ,90-200 Hz (row 3), and 200-300 Hz (row 4) LFP of mouse 2. Red and blue lines represent the swing and stance phases of a step cycle, respectively.

Supplementary Table 1. Electrode Dimension of Different Designs.

Type	Number of Channels	Number of Probes	Channel Number of Each Probe	Electrode Length [μm]	Probe Width [μm]	Thickness [μm]	Electrode Diameter [μm]	Distance between Probe [μm]
SHEA-128	128	4	32	500	100	1	20	300
SHEA-64	64	4	16	680	53	1	30	250

Supplementary Table 2. Immunohistochemistry statistics of SHEA-64

Cell type	Group	Value	Section Number	Comparison group	*p*	df	t statistic
Macrophages	Ctrl[a)]	0.36±0.50	17	Sham vs 2W	0.014	7	-3.25
	2W[b)]	4.49±3.26	11	Sham vs 2M	0.024	7	-2.88
	2M[c)]	1.38±0.50	15	2W vs 2M	0.18	4	1.63
Astrocytes	Ctrl	1.93±0.57	17	Sham vs 2W	1.64×10^{-4}	7	-7.29
	2W	11.25±3.26	11	Sham vs 2M	4.02×10^{-4}	7	-6.31
	2M	4.67±0.71	15	2W vs 2M	0.03	4	3.42
Neurons	Ctrl	13.50±1.33	17	Sham vs 2W	0.18	2	2.00
	2W	7.83±2.45	11	Sham vs 2M	0.38	2	-1.10
	2M	14.97±1.16	14	2W vs 2M	0.0103	4	-4.56
Nuclei	Ctrl	36.97±5.40	17	Sham vs 2W	0.51	2	-0.80
	2W	62.97±28.12	11	Sham vs 2M	0.32	2	-1.30
	2M	55.07±12.01	15	2W vs 2M	0.68	4	0.45

[a)] control group; [b)]2 weeks after implantation; [c)]2 months after implantation.

Supplementary Table 3. Immunohistochemistry statistics of SHEA-128

Cell type	Group	Value	Section Number	Comparison group	*p*	df	t statistic
Macrophages	Ctrl	0.36 ± 0.32	26	Sham vs 2W	$1.52x10^{-4}$	10	-5.89
	2W	3.44±1.63	8	Sham vs 2M	$3.27x10^{-4}$	10	-5.34
	2M	8.11±4.82	7	Sham vs 4M	0.067	10	-2.05
	4M[a)]	0.83 ± 0.43	10	2W vs 2M	0.19	4	-1.59
				2W vs 4M	0.056	4	2.68
				2M vs 4M	0.06	4	2.60
Astrocytes	Ctrl	3.75 ± 1.96	26	Sham vs 2W	$1.03x10^{-4}$	10	-6.19
	2W	11.01±0.41	8	Sham vs 2M	$7.71x10^{-4}$	10	-4.76
	2M	18.24±9.43	7	Sham vs 4M	0.48	10	-0.73
	4M	4.76 ± 2.59	10	2W vs 2M	0.26	4	-1.33
				2W vs 4M	0.015	4	4.12
				2M vs 4M	0.076	4	2.38
Neurons	Ctrl	18.58 ± 8.03	26	Sham vs 2W	0.25	2	1.60
	2W	13.17±2.93	8	Sham vs 2M	0.31	2	1.35
	2M	12.89±3.66	6	Sham vs 4M	0.26	2	1.55
	4M	9.86 ± 4.87	10	2W vs 2M	0.92	4	0.10
				2W vs 4M	0.37	4	1.01
				2M vs 4M	0.44	4	0.86

[a)] 4 months after implantation.

Supplementary Table 4. The thickness of the fluorescent expression around SHEA-64

	Group	Value	Section Number	Comparison group	*p*	df	t statistic
CD68	2W	12.84±5.8	10	2W vs 2M	2.06×10^{-4}	19	4.58
	2M	4.14±2.38	11				
GFAP	2W	15.42±8.62	10	2W vs 2M	0.015	19	2.68
	2M	7.04±5.47	11				

Supplementary Table 5. The thickness of the fluorescent expression around SHEA-128

	Group	Value	Section Number	Comparison group	*p*	df	t statistic
CD68	2W	13.17±5.02	9	2W vs 2M	0.76	14	0.31
	2M	12.47±3.71	7	2W vs 4M	$3.46x10^{-4}$	17	4.46
	4M	4.92+2.88	10	2M vs 4M	$2.63x10^{-4}$	15	-4.74
GFAP	2W	22.16±3.71	9	2W vs 2M	$1.9x10^{-4}$	14	5.01
	2M	14.14±2.26	7	2W vs 4M	$8.33x10^{-7}$	17	7.52
	4M	10.1±3.27	10	2M vs 4M	0.013	15	2.81

Supplementary Table 6. Gait Measures

Time	Stance Width [mm]	Swing Time [ms]	Stride Time [ms]	Gait Symmetry
Pre[a)]	15.50 ± 0.69	120.80 ± 22.41	294.70 ± 73.18	0.98 ± 0.04
Post[b)]1	15.34 ± 1.19	113.40 ± 22.25	292.75 ± 57.73	0.97 ± 0.06
Post3	15.33 ± 0.96	108.283 ± 11.66	257.95 ± 56.66	0.99 ± 0.01
Post7	15.01 ± 0.81	108.95 ± 18.54	244.12 ± 57.68	0.99 ± 0.03

[a)] before SHEA implantation. [b)] days after SHEA implantation

Supplementary Table 7. Parameter comparison between SHEA and other intraspinal electrodes.

Electrode Name	Channel Counts	Target sites	SNR	Units per session	Recording available duration
SHEA	128/64	1200-1400 μm Mouse SC ventral horn	10-30	32 units stable for 3 weeks	1 year
Floating Microelectrode Arrays[c)]	32/13	Marmoset SC	N/A[a)]	N/A[a)]	5.17 months
Carbon Fiber Electrode Arrays[d)]	32	Rat SC dorsal column	10.11 ± 2.09/ 15.62 ± 6.06	N/A[a)]	Few days
carbon nanotube fiber[e)]	2	Rat SC dorsal horn	N/A[a)]	Few units	3-4 months
Nanowire-coated fibers[f)]	1	300 μm Mouse SC	N/A[a)]	Few units	1 week
Braided multi-electrode probes[g)]	22	700-1000 μm Frog SC	4-7	N/A[b)]	5 days
pairs of electromyographic electrodes[h)]	16	1000-2000 μm Rat SC	N/A[a)]	4.4 units	19 days

[a)] Data hasn't been provided in the paper.
[b)] From all sessions and all animals included in this work, they recorded 166 units in total. Units per session can not be calculated from the data.
[c)] N. W. Prins, R. Mylavarapu, A. M. Shoup, S. Debnath, A. Prasad, J Neural Eng 2020, 17 (1), 016031.
[d)] E. Cetinkaya, S. Gok, M. Sahin, Annu Int Conf IEEE Eng Med Biol Soc 2018, 2018, 5069.
[e)] X. Liu, Z. Xu, X. Fu, Y. Liu, H. Jia, Z. Yang, J. Zhang, S. Wei, X. Duan, J Neural Eng 2022, 19 (5).
[f)] C. Lu, S. Park, T. J. Richner, A. Derry, I. Brown, C. Hou, S. Rao, J. Kang, C. T. Mortiz, Y. Fink, P. Anikeeva, Sci Adv 2017, 3 (3), e1600955.
[g)] T. Kim, A. Branner, T. Gulati, S. F. Giszter, J Neural Eng 2013, 10 (4), 045001.
[h)] R. W. Berg, M. T. Chen, H. C. Huang, M. C. Hsiao, H. Cheng, J Neurosci Methods 2009, 182 (1), 49.
